# Supporting people with type 2 diabetes in effective use of their medicine through mobile health technology integrated with clinical care (SuMMiT-D pilot): results of a feasibility randomised trial

**DOI:** 10.1186/s40814-023-01429-5

**Published:** 2024-01-25

**Authors:** The SuMMiT-D Collaborative Group, Andrew J. Farmer, Julie Allen, Y. Kiera Bartlett, Peter Bower, Yuan Chi, David P. French, Bernard Gudgin, Emily Holmes, Robert Horne, Dyfrig A. Hughes, Louise Jones, Cassandra Kenning, Louise Locock, Jennifer McSharry, Lisa Miles, Nicola Newhouse, Rustam Rea, Stephanie Robinson, Lionel Tarassenko, Carmelo Velardo, Nicola Williams, Ly-Mee Yu

**Affiliations:** 1 University of Oxford, Oxford, UK; 2 University of Manchester, Manchester, UK; 3 Patient Representative, Oxford, UK; 4 Bangor University, Bangor, UK; 5 University College London, London, UK; 6 University of Aberdeen, Aberdeen, UK; 7 University of Galway, Galway, Eire Ireland; 8 Oxford University Hospitals NHS Foundation Trust, Oxford, UK; 9 Genomics England, London, UK; 10 Nuffield Department of Primary Care Health Sciences, Woodstock Road, Oxford, OX2 6GG UK

**Keywords:** Type 2 diabetes, Digital health, Behavioural change intervention, Medication adherence, Primary care, Feasibility study, Process evaluation, Randomised controlled trial

## Abstract

**Background:**

The purpose of this 6-month intervention pilot feasibility randomised trial was to test sending brief messages using mobile phones to promote self-management through taking medication as prescribed to people with type 2 diabetes. This was to inform the design and conduct of a future large-scale United Kingdom-based clinical trial and establish the feasibility of recruitment, the technology used, follow-up, and data collection.

**Methods:**

A multicentre individually randomised, controlled parallel group trial in primary care, recruiting adults (≥ 35 years) with type 2 diabetes in England. Consenting participants were randomly allocated to receive short message system text messages up to four times a week, or usual care, for a period of 6 months; messages contained behavioural change techniques targeting medication use. The primary outcome was the rate of recruitment to randomisation of participants to the trial with a planned rate of 22 participants randomised per month. The study also aimed to establish the feasibility of follow-up at 6 months, with an aim of retaining more than 80% of participants. Data, including patient-reported measures, were collected at baseline and the end of the 6-month follow-up period, and a notes review was completed at 24 months.

**Results:**

The trial took place between 26 November 2018 and 30 September 2019. In total 209 participants were randomly allocated to intervention (*n* = 103) or usual care (*n* = 106). The maximum rate of monthly recruitment to the trial was 60–80 participants per month. In total, 12,734 messages were sent to participants. Of these messages, 47 were identified as having failed to be sent by the service provider. Participants sent 2,864 messages to the automated messaging system. Baseline data from medical records were available for > 90% of participants with the exception of cholesterol (78.9%). At 6 months, a further HbA1c measurement was reported for 67% of participants. In total medical record data were available at 6 months for 207 (99.0%) of participants and completed self-report data were available for 177 (84.7%) of participants.

**Conclusion:**

The feasibility of a large-scale randomised evaluation of brief message intervention for people with type 2 diabetes appears to be high using this efficient design. Failure rate of sending messages is low, rapid recruitment was achieved among people with type 2 diabetes, clinical data is available on participants from routine medical records and self-report of economic measures was acceptable.

**Trial registration:**

ISCTRN ISRCTN13404264. Registered on 10 October 2018.

**Supplementary Information:**

The online version contains supplementary material available at 10.1186/s40814-023-01429-5.

## Key messages regarding feasibility

- What uncertainties existed regarding the feasibility?
  While a number of trials evaluating short messaging system (SMS) have been carried out, there has not yet been a large-scale trial in an NHS primary care population using remote recruitment, collection of measures and use of electronic health record data for outcome measures.
- What are the key feasibility findings?
  Recruitment of participants from UK care was achieved at a rate of 60-80 per month and scalable to a larger number of research sites. The SMS messaging system was reliable with only 47 of 12,734 of messages identified as not sent by the service provider. Baseline data from medical records were available for >90% of participants except for cholesterol (78.9%). At 6 months, a further HbA1c measurement was reported for 67% of participants.
- What are the implications of the feasibility findings for the design of the main study?
  The efficient study design used to deliver this trial appears to be feasible with potential for rapid recruitment and efficient follow-up of participants. Further work needs to be carried out prior to a trial to ensure routine data on cholesterol measurements is collected.

## Background

Type 2 diabetes is one of the most common long-term conditions affecting 537 million adults worldwide [1] and 4.7 million people in the UK [2]. It can lead to major complications including cardiovascular disease, renal failure and neuropathy [3]. These complications are preventable [3, 4], but therapy is not always used as recommended, and programmes aimed at self-management do not always engage people with diabetes [5]. Mobile health applications offer novel approaches to addressing these issues.

Medication adherence has been the focus of many mobile health interventions, including the use of brief messages delivered via SMS (short message system) text messages. Low-cost and wide-scale messaging delivered with digital health systems have been shown to be effective in improving health for some conditions and are a promising approach to the problem [6]. Medication adherence interventions are generally considered not very effective and too complex to be widely rolled out [7], in contrast to SMS interventions.

Systematic reviews of text messages to support adherence to treatment, and of other mobile health interventions in diabetes, identify some effective interventions. However, there are many limitations in the studies identified. Of three recent systematic reviews of SMS text messaging to support people with diabetes [8–10], most of the included studies were small (100 participants or less) of short duration (3 to 6 months), focused on people with HbA1c (defined as an HbA1c > 7%) above guideline-recommended levels and compared the intervention to usual care, although usual care varied across studies. Also in these reviews, lack of theory or explicit rationale for SMS content was a major concern [11].

Larger studies and those carried out over a longer period did not generally identify statistically significant differences in measures of HbA1c between intervention and control groups. Two exceptions were a recent study in China that showed a small but significant difference in HbA1c at 6 months in a population with cardiovascular disease and diabetes [12] and a 9-month New Zealand study that included people with type 1 and type 2 diabetes, who were using oral glucose-lowering medication and insulin and the intervention included graphical feedback of blood glucose readings [13].

With evidence for the benefit of brief messaging for type 2 diabetes accumulating, longer trials are needed in a range of health care settings, using systematically developed messages. To address this problem, the SuMMiT-D (SUpport through Mobile Messaging and digital health Technology for Diabetes) programme of work has developed a library of brief messages [14].

These messages (≤ 160 characters in length) are designed to encourage people with type 2 diabetes to develop a habit of taking their medication as intended (that is, to promote effective implementation of dosing and treatment continuation) [15], and provide hints and tips to help them with other aspects of living with the condition.

The current pilot feasibility trial set out to test the feasibility of carrying out a large-scale, effectiveness randomised controlled trial of a mobile phone-based system intended to deliver brief, tailored, behaviour-change messages to a broad and representative range of people with type 2 diabetes focusing on use of medication [16].

The primary objective of the SuMMiT-D Pilot Feasibility trial was to test the recruitment and randomisation of participants to the trial. We tested the proposed primary and secondary outcome data for a randomised controlled trial. We assessed the willingness of participants to be randomised, follow-up rates, data collection for resource use, and trial procedures.

We also carried out work that is or will be reported elsewhere. This included qualitative work with participants and healthcare professionals, and we have included a summary of this work in this paper. We undertook a process evaluation to establish potential mechanisms for the action of the brief messages through changes in hypothesised health psychology constructs [17].

## Methods

### Participants

Participants were recruited from 16 general practices in the Thames Valley and the South Midlands, the West Midlands, the South West Peninsula and the Greater Manchester areas of England from lists of people with type 2 diabetes. Patients eligible for inclusion (aged ≥ 35 years with type 2 diabetes and taking oral glucose-lowering treatment, blood pressure-lowering treatment or lipid-lowering treatment either alone or in combination) were invited to participate in the trial. They needed to have access to a mobile phone and be able, with help (e.g., relative, friend, neighbour) if necessary, to send, understand and retrieve brief SMS text messages in the English language. People using insulin treatment without also using oral glucose-lowering treatment; being pregnant, within 3 months post-partum or planning pregnancy during the trial; having a serious medical condition that made them ineligible; and admission to hospital within the last 3 months for hyper- or hypoglycaemia were excluded.

### Design

**Fig. 1:**
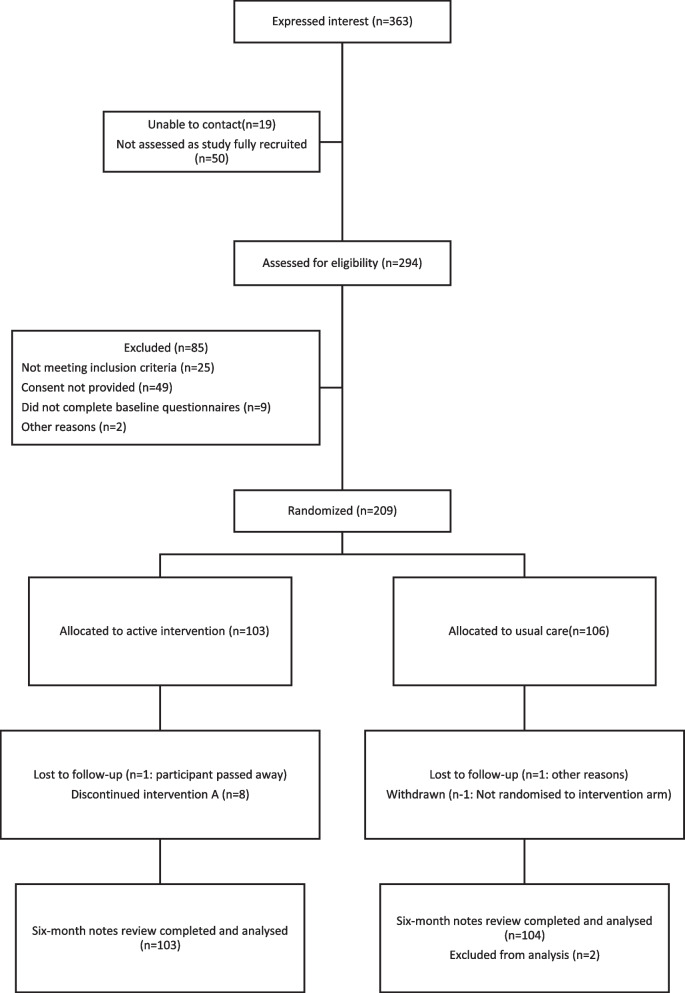
Participant disposition

This study was a primary care-based, two-arm, individually randomised controlled, parallel-group trial done between 26 November 2018 and 30 September 2019 in which eligible patients with type 2 diabetes were randomly allocated to receive Short Messaging System (SMS) text messaging alongside usual care or to usual care (Fig. 1). The trial was carried out in 16 general practices across England.

The study protocol is available as supplementary material (S3) and has been published elsewhere [16]. Ethics approval for the study was obtained from the West of Scotland Ethics Committee 05 (18/WS/0173). All participants were given information about the study and gave electronic or written consent to be included. This study followed the Consolidated Standards of Reporting Trials (CONSORT) reporting guideline [18] and the extension for randomised feasibility and pilot studies [19].

### Intervention

The text messages were developed following a systematic review of the evidence [20], and refined in an iterative process. The SMS messages generated demonstrated acceptability based on patient feedback and fidelity to intended behaviour change determinants, as rated by an independent group of experts [14]. The messages used different behaviour change techniques to target health-related behaviour change relating to the use of medicines. We encouraged participants to seek further relevant information (including the use of links where possible to selected external websites).

Examples of SMS messages used in the trial include: “Plan when, where and how you are going to take your medication”, “It can be difficult to remember to take your tablets. Why not set an alarm to remind you to take them?” and “Visualise in detail how you will take your tablets tomorrow. This will make it easier when you actually take them”.

We undertook further formative work to ensure that the methods of delivering the proposed intervention were appropriate, acceptable and feasible. As a result, we scheduled up to four messages a week at a time of the participants choosing. The frequency of messages received using a particular group of behaviour change techniques could be modified based on a participant's response (through sending back a text message in response to a particular message received). The randomly selected messages were sent by Esendex, an SMS engine provider that operationalised delivery and receipt of text messages between the participants and the automated system. Undelivered messages and messages that were not within the limited range of the system (non-standard) were reviewed regularly. Non-standard messages received a response reminding the participant that the system was not being monitored and giving the research team contact number.

### Measurements

The rate of recruitment of participants to the trial was collected to assess feasibility. We measured recruitment against planned recruitment rates for the proposed main trial, the number of people showing an interest in the trial and not proceeding, and those who withdrew from intervention or trial measurement in both study arms.

To establish the feasibility of collection of clinical and economic measurement data for the proposed main trial we collected data on a range of future trial measures. These included the availability of HbA1c measurements, systolic and diastolic blood pressure, and total to HDL cholesterol ratio data from medical records, and retention to follow-up. Data were obtained from all participants at baseline and 26 weeks after randomisation. We also included data from a 24-month review. Self-reported questionnaires assessed medication adherence using the Medication Adherence Report Scale (MARS) self-report scale [21], health status using the EuroQol 5-Dimension, 5-Level (EQ-5D-5L) scale [22], technology acceptance using a set of measures developed for the study based on the technology acceptance model [23], and healthcare services use (using closed and free text options) to allow healthcare resource use to be costed. A further set of measures assessed the constructs that were targeted by the behaviour change techniques within the messages.

### Study procedures

Potential participants were approached by a letter sent from their general practitioner and those expressing interest by SMS text message were contacted by a researcher who explained the study and screened for eligibility. Eligible patients were sent a link to the online information sheet, consent form and questionnaires. Paper-based measures were available if preferred. Data about clinical outcomes were obtained from a medical notes review. At baseline, we collected demographic data including age, gender, duration of diabetes and medication use. Data on previous use of computers and mobile phones were also collected. Randomisation took place once the baseline assessment was completed with participants allocated to either (i) the intervention programme with participants receiving health-related messages, or (ii) to care as usual with participants only receiving trial-related messages. The randomisation sequence was computer generated using a web-based randomisation programme (Sortition) provided by the University of Oxford Primary Care Clinical Trials Unit. Allocation used a non-deterministic minimisation algorithm to ensure groups were balanced: study site, age (< 65/ ≥ 65 years), gender (male/female), duration of diabetes (< 5 years/ ≥ 5 years), number of medications (< 5/ ≥ 5). Apart from the qualitative research team and the engineering team, the allocation was blinded to all other trial and healthcare staff.

At 26 weeks after randomisation, participants in both groups received SMS messages asking them to complete the follow-up assessments. Reminder messages and phone calls were made if the assessment was not completed. On completion of follow-up, each participant was sent a £10 shopping voucher.

### Statistical analysis

The study was powered to estimate retention to the study over a period of 6 months to ensure that the population recruited through text message would be willing to be followed up. With 200 participants it could estimate 80% follow-up within 95% CIs of 73.8% to 85.3%. The primary outcome was the number of patients recruited to randomisation as a proportion (with 95% confidence interval) of the target recruitment number. Secondary outcomes were reported overall and separately by the allocated arm. Other data are reported as mean or median with standard deviation or Q1, Q3, or as a proportion with percentage.

An exploratory analysis examined the secondary outcomes of the study by allocated arm using available data in the arm to which participants were randomised. Continuous outcomes were analysed with an analysis of covariance adjusted for minimisation factors and results were presented as adjusted differences in means with 95% CI. Binary outcomes were similarly analysed with log-binomial regression models (adjusting for minimisation factors) and results were presented as relative risks and 95% CIs. Healthcare services use questionnaires were assessed for completeness and free-text responses were reviewed to refine the resource use items in the definitive trial.

To inform the development of both the measures of hypothesised determinants of behaviour change and the interview guides for the final trial, a content analysis was conducted of the interviews to identify contextual factors relevant to the intervention and mechanisms by which the intervention may have an effect. The content analysis included data collected in two prior feasibility studies (*n* = 26 and *n* = 38).

## Results

Recruitment to the trial began with the first participant randomised on 26 November 2018 and the last participant randomised on 16 April 2019. The follow-up of participants through medical record data continued until December 2021. Of 363 individuals expressing interest, 19 could not be contacted and 50 had not been assessed as the study was fully recruited. 294 were assessed for eligibility. 85 were excluded (25 did not meet inclusion criteria, 49 did not return a consent form, 9 did not complete baseline questionnaires and two (who had completed paper forms) did not register their mobile phone to take part. 209 participants were randomly allocated to intervention (*n* = 103) or care as usual (*n* = 106) (Fig. 1).

### Participant characteristics

The mean (SD) age of participants was 63.9 (10.2) years (Table 1). The number (%) of women was 86 (41.1%). There were similar proportions of participants above, and below or equal to 65 years. The mean (SD) body mass index (BMI) was 31.6 (6.2). Mean (SD) International Federation of Clinical Chemistry aligned (IFCC) HbA1c was 55.8 (13.5) mmol/mol and for HbA1c Diabetes Control and Complications Trial aligned (DCCT) was 7.3 (1.2)%. Over half of the participants were taking more than five medications a day and nearly three quarters had diabetes for more than 5 years. Of 198 participants with notes review data available, 119 (60.1%) were taking metformin and 140 (70.7%) were taking a statin. All were taking either a glucose-lowering medication (other than insulin), a blood pressure-lowering medication, or a statin.

**Table 1:** Baseline characteristics by randomised group

	SMS messaging (*N* = 103)	Usual care (*N* = 106)	Total (*N* = 209)
	Mean (SD), *n* (%) or Median (Q1, Q3)	Mean (SD) or *n* (%)	Mean (SD) or *n* (%)
Age(years)	63.9 (10.6)	63.9 (9.7)	63.9 (10.2)
Gender—*female*	42 (40.8)	44 (41.5)	86 (41.1)
Height (cm)	171.3 (9.6)	171.3 (9.8)	171.3 (9.7)
Weight (kg)	91.9 (21.7)	93.8 (17.5)	92.9 (19.6)
BMI (kg/m^2^)	31.3 (6.5)	32.0 (5.8)	31.6 (6.2)
Current Smoker	11 (10.7)	5 (4.7)	16 (7.7)
Duration of type 2 diabetes (years)	9.0 (3.2,15.0)	8.2 (4.5, 13.6)	8.8 (4.4, 14.3)
Receiving help with medication	3 (2.9)	3 (2.8)	6 (2.9)
Taking ≥ 5 medications (self-reported)	60 (58.3)	59 (55.7)	119 (56.9)
Ethnic group			
*White British*	88 (85.4)	91 (85.8)	179 (85.6)
*Indian*	2 (1.9)	4 (3.8)	6 (2.9)
*Pakistani*	3 (2.9)	1 (0.9)	4 (1.9)
*Caribbean*	0 (0.0)	2 (1.9)	2 (1.0)
*Chinese*	1 (1.0)	0 (0.0)	1 (0.5)
*African*	0 (0.0)	1 (0.9)	1 (0.5)
*Any mixed/multiple ethnic background*	1 (1.0)	1 (0.9)	2 (1.0)
*Other White background*	4 (3.9)	2 (1.9)	6 (2.9)
*Other South Asian*	1 (1.0)	0 (0.0)	1 (0.5)
*Other White British background*	0 (0.0)	1 (0.9)	1 (0.5)
*Other Asian*	0 (0.0)	1 (0.9)	1 (0.5)
*Other Black/African/Caribbean background*	1 (1.0)	0 (0.0)	1 (0.5)
*Other*	1 (1.0)	0 (0.0)	1 (0.5)
Education group—*n* (%)			
*Some secondary education*	20 (19.4)	4 (3.8)	24 (11.5)
*GCSE/O-Levels*	22 (21.4)	29 (27.4)	51 (24.4)
*College, A-Levels, NVQ3 or below*	15 (14.6)	24 (22.6)	39 (18.7)
*Diploma, certificate, BTEC, NVQ 4 and above*	15 (14.6)	21 (19.8)	36 (17.2)
*Undergraduate degree (BA, BSc)*	14 (13.6)	18 (17.0)	32 (15.3)
*Post-graduate degree (MA, MSc)*	13 (12.6)	7 (6.6)	20 (9.6)
*Doctorate (PhD)*	0 (0.0)	1 (0.9)	1 (0.5)
HbA_1c_ (mmol/mol)	56.5 (14.7)	55.1 (12.3)	55.8 (13.5)
HbA_1c_ (%)	7.3 (1.3)	7.2 (1.1)	7.3 (1.2)
Total cholesterol (mmol/L)	3.9 (0.9)	3.8 (0.7)	3.9 (0.8)
HDL cholesterol (mmol/L)	1.4 (0.7)	1.3 (0.5)	1.4 (0.6)
Ratio of total to HDL cholesterol	3.2 (1.3)	3.2 (1.0)	3.2 (1.2)
Systolic blood pressure (mmHg)	130.9 (13.4)	133.2 (14.6)	132.0 (14.1)
Diastolic blood pressure (mmHg)	76.3 (9.1)	76.2 (8.9)	76.2 (9.0)
Previous myocardial infarction	6 (5.8)	7 (6.6)	13 (6.2)
Stroke	1 (1.0)	2 (1.9)	3 (1.4)
Previous transient ischemic attack	1 (1.0)	3 (2.8)	4 (1.9)
Peripheral vascular disease	3 (2.9)	3 (2.8)	6 (2.9)
Renal Failure	2 (1.9)	1 (0.9)	3 (1.4)
Period owned or used a mobile phone			
*One year or less*	0 (0.0)	1 (0.9)	1 (0.5)
*One to 2 years*	0 (0.0)	1 (0.9)	1 (0.5)
*Two to 5 years*	7 (6.8)	7 (6.6)	14 (6.7)
*More than 5 years*	95 (92.2)	97 (91.5)	192 (91.9)
Type of mobile phone			
*Smartphone*	89 (86.4)	91 (85.8)	180 (86.1)
*Standard mobile phone*	14 (13.6)	15 (14.2)	29 (13.9)
EQ-5D-5L VAS^a^	74.4 (19.9)	75.2 (15.2)	74.8 (17.6)
EQ-5D-5L Index^b^	0.8 (0.2)	0.8 (0.2)	0.8 (0.2)
MARS^c^	22.9 (2.7)	23.7 (1.8)	23.3 (2.3)

^a^Score ranges from 0 to 100, where 0 represents the “worst health you can imagine” and 100 represents the “best health you can imagine”

^b^Range from less than 0 (where 0 is the value of a health state equivalent to dead; negative values representing values as worse than dead) to 1 (the value of full health), with higher scores indicating higher health utility

^c^Medication adherence rating scale (five items). Score ranges from 5 to 25, where higher scores indicate better adherence

### Recruitment

**Fig. 2:**
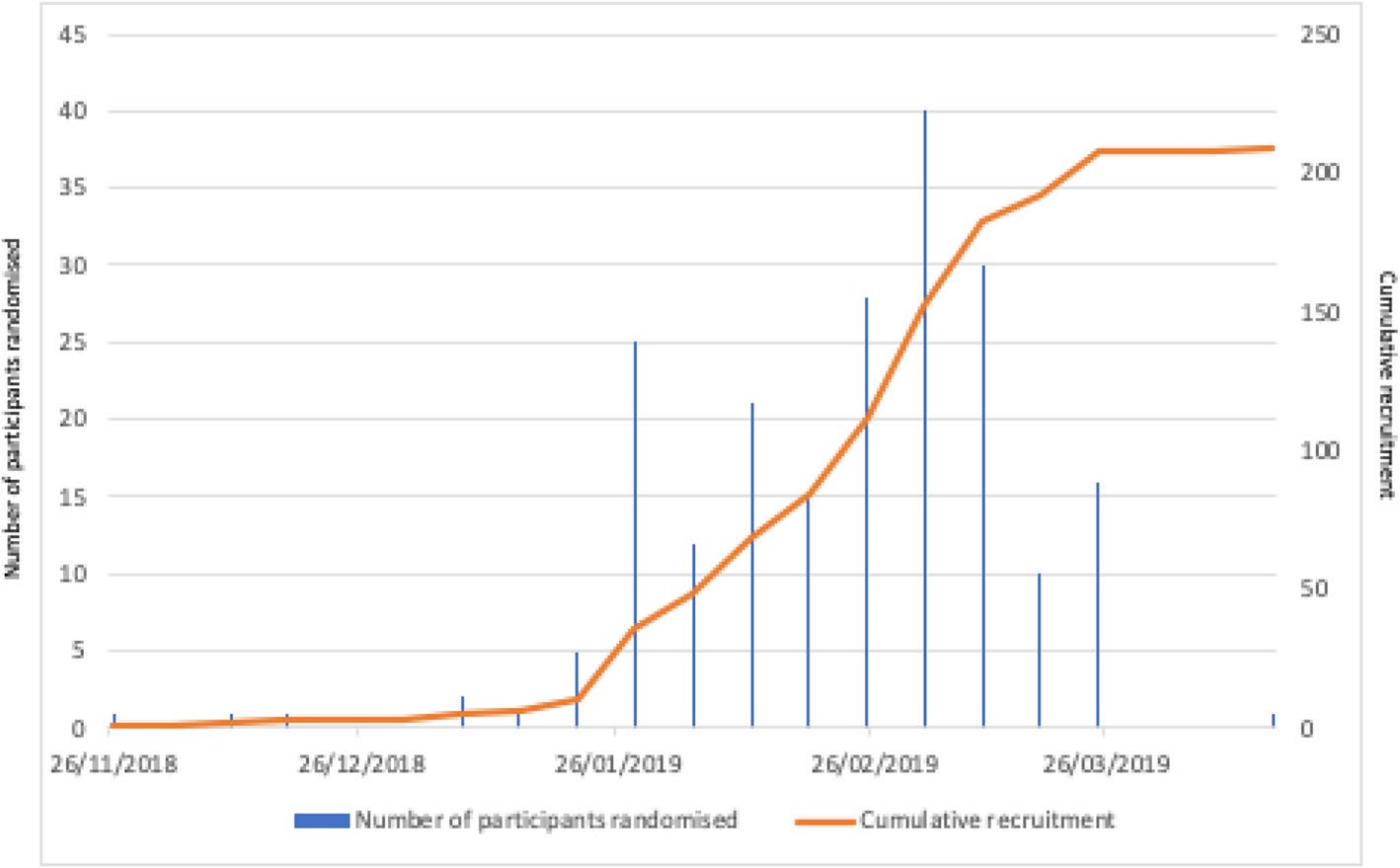
Recruitment of participants over time

Recruitment started slowly over the month of December (Fig. 2). However, once recruitment had started an average of 22 participants each week were randomised (Fig. 2) and exceeded the proposed sample size of 200 within 4 months of the first participant recruited (the original planned recruitment period was 9 months).

### Loss to follow-up and withdrawal

Loss to follow-up and withdrawal from the trial are reported in Fig. 1. One participant (allocated to active intervention) died shortly after randomisation and was withdrawn from the analysis, and one participant was allocated to usual care but withdrawn. Participant-completed questionnaires were received from all participants prior to randomisation. Eight participants in the SMS intervention group and two in the usual care group discontinued the intervention. Four participants from the intervention arm who asked to stop receiving messages commented that they did not consider the messages helpful or relevant to them. For the exploratory follow-up analysis, one further participant in the intervention group was lost to follow-up having moved practice. Four further participants were lost from the usual care group. In total, health record data were available for 207 (99.0%) participants.

### Collection of clinical data

The baseline clinical data collected are reported in Table 1. The manual extraction of electronic health records provided comprehensive data on participants. Data were available for HbA1c, weight and blood pressure within a mean (SD) number of days of randomisation of 167 (79), 153 (100) and 131 (90), respectively. Cholesterol appeared to be measured less frequently with the nearest measure (mean, SD) prior to randomisation of 217 (107) days.

A notes review was carried out at 24 months to explore potential problems with collecting longer-term data and to provide material for an exploratory analysis of outcomes with data collected for an eighteen-month endpoint (Supplementary Tables 1 and 2).

### Collection of self-reported data

Collection of self-reported data was found to be feasible and acceptable. Data are reported in Table 1, and the proportion of missing data (Table 2) was very low. One hundred sixty (77%) participants completed self-report questionnaires on-line, and 49 (23%) completed paper copies of the questionnaires and returned them by post. Reporting of hypothesised mediators of behaviour change are reported elsewhere [17].

**Table 2:** Baseline data completeness by randomised group

Variable, *n* (%)	SMS messaging (*N* = 103)	Usual care (*N* = 106)	Total (*N* = 209)
*Notes review*			
Height	97 (94.2)	96 (90.6)	193 (92.3)
HbA1c	101 (98.1)	99 (93.4)	200 (95.7)
Total cholesterol	87 (84.5)	78 (73.6)	165 (78.9)
HDL cholesterol	96 (93.2%)	93 (87.7%)	189 (90.4%)
Blood pressure	103 (100.0)	104 (98.1)	207 (99.0)
Weight	100 (97.1)	102 (96.2)	202 (96.7)
MI	103 (100.0)	104 (98.1)	207 (99.0)
Stroke	103 (100.0)	104 (98.1)	207 (99.0)
TIA	103 (100.0)	104 (98.1)	207 (99.0)
Heart failure	103 (100.0)	104 (98.1)	207 (99.0)
PVD	103 (100.0)	104 (98.1)	207 (99.0)
Renal failure	103 (100.0)	104 (98.1)	207 (99.0)
Smoking status	101 (98.1)	105 (99.1)	206 (98.6)
*Self-reported questionnaires*			
EQ-5D-5L^1^	103 (100.0)	105 (99.1)	208 (99.5)
MARS^1,2^	97 (94.2)	100 (94.3)	197 (94.3)
Health care psychology^3^	103 (100.0)	106 (100.0)	209 (100.0)
Health services use^3^	103 (100.0)	106 (100.0)	209 (100.0)

^1^Number (%) indicates the number of participants who returned questionnaires and all items were completed

^2^Medication adherence rating scale (5 items)

^3^Number (%) represents the number of participants who returned the questionnaire

### Feasibility and acceptability of the intervention

The delivery of the messages was found to be feasible and the content acceptable to participants. Factors moderating acceptability included participant context, such as duration of condition, co-morbidities, level of understanding of diabetes management, stability of existing routines, support from others and sense of self as a person with diabetes. System factors such as perceived message tone, novelty, relevance and clarity were also important moderators. For example, messages phrased as rhetorical questions prompted some participants to respond in full, triggering an error message from the automated system. Some participants who lived alone or who experienced limited support from others described messages which encouraged family involvement in diabetes management as upsetting. Other participants who had stable adherence routines in place described the practical tips intended to support habit formation to be patronising. Previous use of digital tools was not perceived as advantageous, and messages were acceptable to participants using feature mobile phones (i.e. not smart devices). The qualitative findings relating to the feasibility and acceptability of the messages will be reported in full elsewhere.

### Medication data

Collecting prescribing data was not feasible for this feasibility study because of the difficulties in working with practices to collect the volume of data. This is addressed in the discussion, and solutions have been identified. Self-report of medication use in the past month was recorded by patients within the healthcare resource use questionnaire. The feasibility of costing electronic prescribing data was tested using sample data, to assess required data fields and costing techniques; and to ensure efficient collection of prescription cost data in the definitive trial.

### Follow-up

Clinical data was collected from review of medical records at 6 months for all participants, with new HbA1c data available for 140 (67%) participants. New blood pressure, weight and cholesterol measures were available in an additional proportion of participants, but using retrospective data, the mean (SD) interval between HbA1c measurements was 100 (37.9) days, total cholesterol 99 (50.6) days, blood pressure 44 (42) days and weight 60 (45.5) days. Review of clinical records identified inconsistency in identification of lipid measurements in the clinical record with differing codes used from different laboratories. Data on new diabetes complications were not collected at 6-month follow-up because of the short period of follow-up. In total medical record data were available for 207 (99.0%) study participants.

Completed self-report data were received from 177 (84.7%) of participants and is included below. Free-text responses on the healthcare resource use questionnaire were reviewed to refine the questionnaire, reporting of primary care consultation type use was most common (diabetes nurse, health care assistant, phlebotomist). Data on the changes in beliefs observed in this trial have been reported separately [17].

### Exploratory analysis of outcomes

Analysis of available data for clinical parameters at 6 months and eighteen months are reported in Supplementary Tables 1 and 2. No clinically important changes from baseline were observed.

### Number of messages sent/failed

In total, 12,734 messages were sent to participants. Of these messages, 47 were identified as having failed to be sent by the service provider. Participants sent 2864 messages to the automated messaging system.

## Discussion

This study of brief, tailored, behaviour-change messages to people with type 2 diabetes focusing on the use of medication appears feasible to deliver and acceptable to participants. The study design was a randomised trial using an electronic and automated data management system integrating trial sign-up, data collection and message delivery. The option of postal questionnaires was requested by 23% of trial participants. Rates of data completion using the web-based system were high. The use of a sign-up system based on sending a text message led to a high rate of conversion to screening and subsequent randomisation. Only 78 of 364 patients expressing interest did not respond at subsequent steps in the trial recruitment process.

Review of records indicated that over half of the participants would be likely to have clinical data collected within 6 months of the trial endpoint. We identified the need to revise our protocol for identifying lipid measurement codes in clinical records to capture all the available data.

These data were collected during the period when Covid impacted clinical care which impacted on availability of clinical data where routine checks were delayed. To reduce the risk of missing data in a future trial we propose to obtain permission from participants to directly access electronic clinical record data, and to support practices in identifying where individuals have not had clinical measurements recorded in line with recommended care guidelines.

The system used to send messages had a very low rate for message delivery failure. Message delivery failure arose from changing phone number, and phone not being used because of illness or extended overseas travel. Troubleshooting to resolve these issues was straightforward.

Technically the intervention was feasible to deliver at scale. The system demonstrated an efficient design for delivering messages and for carrying out the trial. The high levels of exposure to messages were confirmed by comments in post-trial interviews. More detailed reports of qualitative work are currently in preparation.

The exploratory analysis of clinical measurements identified the limitations of collecting data manually from the clinical records. The trial was not designed to establish a signal of efficacy.

A process evaluation looking at reported changes in the way that the messages appear to change hypothesised determinants of medication taking has confirmed that the intervention appears to modify psychological constructs and that changes in psychological constructs are associated with changes in medication taking [17]. This provides an additional signal that the instance of SMS messaging to be evaluated in the clinical trial has an underpinning evidence base.

The main outcomes of this study were to confirm the feasibility and acceptability of the intervention and the proposed substantive trial. Specific changes, building on this study that will be needed for a substantive trial include further redrafting of participant information leaflets, additional simplification of study procedures to streamline the process of recruitment to the study and editing the qualitative interview guide and the quantitative measures to ensure we gather further information related to contextual factors and potential mechanisms. Self-reported healthcare resource use was acceptable and relevant, the findings of the pilot feasibility study have informed modification of the questionnaire to reduce burden and improve engagement. Manual extraction of data from clinical records proved challenging. Building on the experience of the trial a future study will implement the use of primary care electronic record data, with appropriate governance, consent and secure storage of pseudonymised data to improve the efficiency and accuracy of the clinical data used.

## Limitations

As with many similar trials, only a small proportion of people with diabetes contacted by their primary care team expressed interest in taking part in the study. Nonetheless, those taking part in the trial did not appear to have extensive experience of using digital interventions.

The lower age threshold of 35 years for trial participants was a pragmatic approach to avoid including larger numbers of people with possible type 1 diabetes, and because the focus of this work was on people who might be less familiar with mobile phones.

This study was carried out at the time the General Data Protection Regulations were introduced. This led to delays in approvals of the study. However, the experience of doing this work in the context of changes in regulation allowed rapid progression of the design of a substantive trial.

Self-reported medication adherence data with a 1-month retrospective review may be subject to recall bias, but this bias would apply to both control and intervention groups. More frequent collection of adherence data with diary or daily messaging was considered by our patient advisory group to be intrusive.

Manual extraction of data from clinical records proved challenging. Review of data suggested that some parameters, specifically levels of cholesterol, were not correctly identified or recorded despite piloting data extraction forms and training staff.

## Conclusions

Sending regular health-related SMS text messages to people with type 2 diabetes is feasible. Evaluation in clinical trials is also feasible using an efficient design. A future trial could recruit sufficient participants to test whether the SMS-based brief messaging system provides clinical benefit, and if so, to what extent, compared to usual care. Further evaluation in a large-scale pragmatic trial is needed to test the extent of benefit and whether this type of intervention offers value for money.

### Supplementary Information

**Additional file 1. **CONSORT 2010 checklist of information to include when reporting a pilot or feasibility trial.

**Additional file 2. **Supplementary results tables and figures. Table S1 and S2. Six- and eighteen-month analysis of change in clinical parameters. Feasibility Trial Protocol. **Supplementary Table 1.** Change in clinical parameters baseline to a window of 3-12 months, whichever is closer to six months with available clinical data. **Supplementary Table 2.** Change in clinical parameters baseline to a window of 12-24 months, whichever is closer to eighteen months with available clinical data. **Supplementary Table 3.** Trial measurement and study objectives.

**Additional file 3. **

## Data Availability

Qualifying researchers who wish to access our data should submit a proposal with a valuable research question. Proposals will be assessed by a committee formed from the trial management group, including senior statistical and clinical representation. Data will be shared in accordance with the data sharing policy of Nuffield Department of Primary Care Health Sciences.
