# Supplementary material for: Supporting people with type 2 diabetes in effective use of their medicine through mobile health technology integrated with clinical care (SuMMiT-D pilot): results of a feasibility randomised trial
Source: Pilot Feasibility Stud. 2024 Jan 25;10:15. doi: 10.1186/s40814-023-01429-5 (PMC10809651; doi:10.1186/s40814-023-01429-5)
Supplement: Supplementary file 2 — Additional file 2. Supplementary results tables and figures. Table S1 and S2. Six- and eighteen-month analysis of change in clinical parameters. Feasibility Trial Protocol. Supplementary Table 1. Change in clinical parameters baseline to a window of 3-12 months, whichever is closer to six months with available clinical data. Supplementary Table 2. Change in clinical parameters baseline to a window of 12-24 months, whichever is closer to eighteen months with available clinical data. Supplementary Table 3. Trial measurement and study objectives. [file 40814_2023_1429_MOESM2_ESM.docx]

Supplementary Data

Message delivery

## Supplementary material

Table S1 and S2: Six- and eighteen-month analysis of change in clinical parameters

Feasibility Trial Protocol

## Supplementary data

### Supplementary Table 1: Change in clinical parameters baseline to a window of 3-12 months, whichever is closer to six months with available clinical data.

| Outcome | Mean change from baseline to 6 months (SD)  n | | Adjusted mean difference between groups (95% CI)* (Mean in Intervention minus mean in Control) |
| --- | --- | --- | --- |
|  | Intervention | Control |  |
| HbA1c (%) | -1.13 (12.86)  79 | -1.66 (10.54)  75 | 1.64 (-1.66 to 4.94) |
| HDL Cholesterol Mmol/L | -0.20 (0.70)  69 | -0.10 (0.49)  57 | -0.05 (-0.15 to 0.04) |
| Total Cholesterol Mmol/L | 0,15 (0.99)  41 | 0.11 (0.46)  25 | 0.10 (-0.29 to 0.49) |
| Systolic Blood Pressure mmHg | -0.83 (15.0)  82 | -1.14 (12.60)  85 | -0.23 (-4.06 to 3.60) |
| Diastolic Blood Pressure mmHg | -0.18 (9.13)  82 | 0.39 (10.79)  85 | -0.29 (-2.75 to 2.17) |

### Supplementary Table 2: Change in clinical parameters baseline to a window of 12-24 months, whichever is closer to eighteen months with available clinical data.

| Outcome | Mean change from baseline to 18 months (SD)  n | | Adjusted mean difference between groups (95% CI)* (Mean in Intervention minus mean in Control) |
| --- | --- | --- | --- |
|  | Intervention | Control |  |
| HbA1c (%) | 0.88 (14.15)  77 | 0.28 (12.51)  80 | 1.47 (-2.25 to 5.19) |
| HDL Cholesterol Mmol/L | -0.20 (0.75)  69 | -0.07 (0.49)  68 | -0.08 (-0.19 to 0.03) |
| Total Cholesterol Mmol/L | -0.01 (0.86)  34 | 0.11 (0.52)  31 | 0.04 (-0.26 to 0.35) |
| Systolic Blood Pressure mmHg | -0.55 (16.62)  71 | -0.12 (18.55)  78 | -2.03 (-6.63 to 2.57) |
| Diastolic Blood Pressure mmHg | 1.8 (10.42)  71 | -1.44 (10.54)  78 | 3.30 (0.66 to 5.94) |

### Supplementary Table 3 Trial measurement and study objectives

| **Trial variable** | **Research question(s)** |
| --- | --- |
| - Date of randomisation | 1,2 |
| - Date of follow up | 1,2 |
| - Numbers of SMS messages sent, identified as not received, failed | 2 |
| **Self-report variables** |  |
| - MARS self-report scale (Questions about using your medicines) | 3 |
| - EQ-5D-5L (Health status EQ-5D-5L) | 3 |
| - Health psychology theory and the technology acceptance model. (Ideas and concerns about your diabetes treatment) | 3,4 |
| - Healthcare utilisation record (Health services use) | 3,5 |
| - Experience of diabetes education | 6 |
| - Presence of a carer and their role in dose administration | 3 |
| - Duration of diabetes | 3 |
| - Self-reported level of education | 6 |
| - Smoking status | 3 |
| - Age | 6 |
| - Gender | 6 |
| - Ethnicity | 6 |
| - Current mobile phone use. | 6 |
| **Variables collected from electronic health record** |  |
| - HbA1c, | 3 |
| - Systolic and diastolic blood pressure, | 3 |
| - Total and HDL cholesterol level, | 3 |
| - Weight | 3 |
| - Height | 6 |
| - Current medication and prescriptions for diabetes drugs (glucose lowering, blood pressure lowering and lipid lowering) | 6 |
| - Previous occurrence of myocardial infarction, stroke, Transient Ischemic Attack (TIA), heart failure, peripheral vascular disease, and renal failure | 6 |

1. Feasibility of recruitment, randomisation and follow up of participants.
2. Feasibility of trial procedures.
3. Feasibility of collection of proposed primary and secondary outcome data.
4. Hypothesised health psychology constructs.
5. Data collection for resource use.
6. Mediating or moderating variables
