# Supplementary material for: Supporting people with type 2 diabetes in effective use of their medicine through mobile health technology integrated with clinical care (SuMMiT-D pilot): results of a feasibility randomised trial
Source: Pilot Feasibility Stud. 2024 Jan 25;10:15. doi: 10.1186/s40814-023-01429-5 (PMC10809651; doi:10.1186/s40814-023-01429-5)
Supplement: Supplementary file 3 — Additional file 3. [file 40814_2023_1429_MOESM3_ESM.pdf]

Trial Title: Supporting people with type 2 diabetes in effective use of their medicine through a system comprising mobile health technology integrated with clinical care compared with usual care: a randomised feasibility trial

Internal Reference Number / Short title: SuMMiT-D Feasibility: **SU**pport through **M**obile **M**essaging and digital health **T**echnology for **D**iabetes / SuMMiT-D: Feasibility

Ethics Ref: 18/WS/0173

Date and Version No: 23 August 2018 v1.0

Chief Investigator:

Professor Andrew Farmer
Nuffield Department of Primary Care Health Sciences
University of Oxford
Radcliffe Observatory Quarter
Woodstock Road
Oxford OX2 6GG
Tel: +44 (0)1865 617942

Investigators:

Prof Peter Bower, University of Manchester
Prof David French, University of Manchester
Prof Dyfrig Hughes, Bangor University
Prof Louise Locock, University of Aberdeen
Prof Lionel Tarassenko, University of Oxford
Dr Kiera Bartlett, University of Manchester
Dr Jennifer McSharry, National University of Ireland, Galway
Dr Carmelo Velardo, University of Oxford
Dr Veronika Williams, University of Oxford
Dr Ly-Mee Yu, University of Oxford
Nikki Newhouse, University of Oxford

Trial Management:

Julie Allen, University of Oxford
Evgenia Riga, University of Oxford

Sponsor:

University of Oxford
Joint Research Office
1st Floor, Boundary Brook House
Churchill Drive, Headington
Oxford OX3 7LQ

Funder:

National Institute for Health Research (NIHR) Programme Grant for Applied Research Ref: RP-PG-1214-20003

Chief Investigator Signature:

Statistician Signature:

The authors declare there are no potential conflicts of interest.

Confidentiality Statement

This document contains confidential information that must not be disclosed to anyone other than the Sponsor, the Investigator Team, HRA, host organisation, and members of the Research Ethics Committee, unless authorised to do so.

© Copyright: The University of Oxford and Oxford University Hospitals NHS Foundation Trust 2016

KEY TRIAL CONTACTS

**Chief Investigator**	Professor Andrew Farmer<br>Nuffield Department of Primary Care Health Sciences<br>University of Oxford, Radcliffe Observatory Quarter,<br>Woodstock Road<br>Oxford, OX2 6GG<br>**Tel:** +44 (0)1865 617942<br>**Fax:** +44 (0)1865 289412<br>**Email:** andrew.farmer@phc.ox.ac.uk
**Sponsor**	Ms Heather House<br>Clinical Trials and Research Governance, University of Oxford<br>Joint Research Office,<br>1st Floor, Boundary Brook House,<br>Churchill Drive, Headington, Oxford, OX3 7LQ<br>**Email:** ctrg@admin.ox.ac.uk
**Clinical Trials Unit**	Primary Care Clinical Trials Unit,<br>Nuffield Department of Primary Care Health Sciences<br>University of Oxford, Radcliffe Primary Care<br>Radcliffe Observatory Quarter, Woodstock Road<br>Oxford, OX2 6GG
**Statistician**	Associate Professor Ly-Mee Yu<br>Nuffield Department of Primary Care Health Sciences<br>University of Oxford, Radcliffe Primary Care<br>Radcliffe Observatory Quarter, Woodstock Road<br>Oxford, OX2 6GG<br>**Tel:** +44 (0)1865 617199<br>**Fax:** +44 (0)1865 289412<br>**Email:** ly-mee.yu@phc.ox.ac.uk

© Copyright: The University of Oxford and Oxford University Hospitals NHS Foundation Trust 2016
CONFIDENTIAL

I SYNOPSIS

**Study Title**	Supporting people with type 2 diabetes in effective use of their medicine through a system comprising mobile health technology integrated with clinical care compared with usual care: a randomised feasibility trial	
**Internal ref. no. (or short title)**	**SuMMiT-D Feasibility**: **SU**pport through **M**obile **M**essaging and digital health **T**echnology for **D**iabetes / SuMMiT-D: Feasibility	
**Study Design**	Multicentre randomised controlled feasibility trial	
**Study Participants**	Cohort 1 - Patients aged ≥35 years with type 2 diabetes<br>Cohort 2 – Primary care health professionals	
**Planned Sample Size**	Cohort 1 - We aim to recruit 200 patients<br>Cohort 2 - We aim to recruit between 12 and 30 healthcare professionals based on considerations of data saturation	
**Follow up duration**	Two years	
**Planned Study Period**	01/09/ 2018 to 31/08/ 2021	
	**Objectives**	**Outcome Measures**
**Primary**	To test participant recruitment to trial and willingness to be randomised.	To measure recruitment against planned recruitment rates.<br><br>Number of people showing an interest and not proceeding or those who withdraw from the control group and give a reason
**Secondary**	i. To assess the feasibility of collection of clinical measurement data for the proposed clinical trial. (With particular interest in prior to entry or following trial entry measurement collection of participant's HbA1c, systolic blood pressure and cholesterol).<br><br>ii. To test willingness of participants to be followed up over the 26-week period post randomisation.<br><br>iii. To test the collection of prescribing data on trial participants.<br><br>iv. To test the collection of self-reported questionnaire measures.	i. Completeness of data collection of HbA1c, systolic blood pressure and total to HDL cholesterol ratio.<br><br>ii. Retention and follow-up rates.<br><br>iii. Proportion of medication possession ratio for glucose, blood pressure and lipid lowering medication obtainable.<br><br>iv. Proportions of completed self-reported measures.

© Copyright: The University of Oxford and Oxford University Hospitals NHS Foundation Trust 2016

	v. To assess the feasibility and acceptability of the intervention for patients and healthcare professionals (including general practitioners, nurses, receptionists and pharmacists).<br><br>vi. To assess and understand the mechanisms of action of brief health related messages on self-reported adherence.<br><br>vii. To assess feasibility and acceptability of self-completion of the resource use questionnaire.	v. Data obtained through focus groups/qualitative interviews with patients and recruiting healthcare staff.<br><br>vi. Change in quantitative process measures and relationship between changes in these measures and self-reported adherence.<br><br>vii. Proportions of completed resource use questionnaire.
**Exploratory**	viii. Changes in primary and secondary measures that will be used in the main trial<br><br>ix. Information on message delivery and interaction with participants.	viii. Differences between time points obtained from baseline to six months.<br><br>ix. Automated reports from messaging service on messages delivered and interactive messaging
**Intervention**	Brief, tailored behaviour change messages delivered to people with type 2 diabetes focusing on use of medication using a mobile phone based system.	

© Copyright: The University of Oxford and Oxford University Hospitals NHS Foundation Trust 2016

II ABBREVIATIONS

AE	Adverse Event
AR	Adverse Reaction
BCT	Behaviour Change Technique
CI	Chief Investigator
CRF	Case Report Form
CTRG	Clinical Trials and Research Governance
DMC	Data Monitoring Committee / Data Monitoring and Safety Committee
GP	General Practitioner
GCP	Good Clinical Practice
HbA1c	Glycated Haemoglobin
HRA	Health Research Authority
ICH	International Conference on Harmonisation
ICMJE	International Committee of Medical Journal Editors
ISRCTN	International Standard Randomised Controlled Trials Number
MARS	Medication Adherence Rating Scales
NHS	National Health Service
NIHR	National Institute for Health Research
PC-CTU	Primary Care Clinical Trials Unit
PIL	Participant Information Leaflet
PSC	Programme Steering Committee
REC	Research Ethics Committee
SAE	Serious Adverse Event
SAR	Serious Adverse Reaction
SMS	Short Message Service
SOP	Standard Operating Procedure
SUSAR	Suspected Unexpected Serious Adverse Reactions
TIA	Transient Ischemic Attack
TMG	Trial Management Group

© Copyright: The University of Oxford and Oxford University Hospitals NHS Foundation Trust 2016

Table of Contents

KEY TRIAL CONTACTS 3
I SYNOPSIS 4
II ABBREVIATIONS 6
TABLE OF CONTENTS 7
1 BACKGROUND AND RATIONALE 10
1.1 Disease area 10
1.2 Evidence from systematic reviews 10
1.3 Preliminary data 10
1.4 Aims of current study 10
2 OBJECTIVES, STUDY PARAMETERS AND ENDPOINTS 11
2.1 Summary of objectives and outcomes 11
2.2 Aims and objectives of the SuMMiT-D feasibility study 12
2.2.1 Primary outcome 12
2.2.2 Secondary outcomes 12
3 TRIAL DESIGN 13
3.1 Intervention study 13
3.2 Process evaluation 13
3.3 Treatment of participants 14
3.3.1 Tailored mobile-phone messaging system 14
3.3.2 Usual care 14
3.3.3 Potential risks and benefits of the tailored mobile-phone messaging system 14
3.4 PARTICIPANT IDENTIFICATION 15
3.4.1 Trial Participants 15
3.4.2 Inclusion Criteria 15
3.4.3 Exclusion Criteria 15
3.5 TRIAL PROCEDURES 15
3.5.1 Recruitment procedures 16
3.5.2 Screening Assessment 16
3.6 Informed Consent 17
3.7 Randomisation, blinding and code-breaking 17
3.8 Data Collection .... **Error! Bookmark not defined.**
3.8.1 The following procedures will take place for the baseline assessment: 18
3.8.2 Follow-up (data collection) 19
3.9 Long term follow up 19

© Copyright: The University of Oxford and Oxford University Hospitals NHS Foundation Trust 2016

3.10 Discontinuation or withdrawal of participants from trial ... 19
3.11 Definition of End of Trial ... 20
4 Process analysis ... 20
4.1 Qualitative work with study participants ... 20
4.2 Focus Group Work with primary care staff ... 20
5 SAFETY REPORTING ... 21
5.1 Definitions ... 21
5.2 Causality ... 22
5.3 Procedures for Recording Adverse Events ... 23
5.4 Reporting Procedures for Serious Adverse Events ... 23
6 STATISTICS ... 24
6.1 Description of Statistical Methods ... 24
6.2 The Number of Participants ... 24
7 DATA MANAGEMENT ... 24
7.1 Source Data ... 24
8 Access to Data ... 25
8.1 Data Recording and Record Keeping ... 25
9 QUALITY ASSURANCE PROCEDURES ... 25
9.1 Trial Management Group ... 26
9.2 Trial Steering and Data Monitoring Committees ... 26
10 ETHICAL AND REGULATORY CONSIDERATIONS ... 26
10.1 Declaration of Helsinki ... 26
10.2 Guidelines for Good Clinical Practice ... 26
10.3 Approvals ... 26
10.4 Reporting ... 26
10.5 Participant Confidentiality ... 27
10.6 Expenses and Benefits ... 27
10.7 Other Ethical Considerations ... 27
11 FINANCE AND INSURANCE ... 27
11.1 Funding ... 27
11.2 Insurance ... 27
12 PUBLICATION POLICY ... 27
13 REFERENCES ... 28
14 APPENDIX A: TRIAL FLOW CHART ... 29
15 APPENDIX B: SCHEDULE OF PROCEDURES TABLE & TIMELINE ... 30

© Copyright: The University of Oxford and Oxford University Hospitals NHS Foundation Trust 2016

16 APPENDIX C: INTERVENTION COMPONENTS 33
16.1 Engineering: SuMMiT-D Feasibility backend SMS system........ 33
16.2 Health Psychology 33
17 APPENDIX D: AMENDMENT HISTORY........ 37

© Copyright: The University of Oxford and Oxford University Hospitals NHS Foundation Trust 2016

1 BACKGROUND AND RATIONALE

1.1 Disease area

Type 2 diabetes is a lifelong condition that causes a person's blood sugar to become too high. It can cause serious long-term health problems. 3.4 million people in the UK are affected [1]. Treatments to reduce risks of complications from type 2 diabetes are effective if taken as intended. Concerns about medicines and difficulties in taking them regularly, whether intentional or unintentional, are common. Some services to help are available, but evidence of their effectiveness and cost-effectiveness is weak. These services may not be right for everyone and are not designed for repeated use. Not everyone has access to this extra help.

In addition to the burden of preventable death and disability, the cost of non-adherence with diabetes treatments has been estimated at £100 million per year in avoidable treatment costs. [2] Understanding and improving this situation could make a major contribution to health and NHS costs. At the moment managing the cost of diabetes complications takes up 10% of the NHS budget.

1.2 Evidence from systematic reviews

Systematic reviews do not provide strong evidence to support the wider use of current approaches to developing interventions to support adherence.[3] Brief messages delivered at a wide-scale and low cost via digital health systems added to usual care have been shown to be effective in improving health for some conditions and are a promising approach to the problem.[4]

Systematic reviews of text-messaging to support adherence to treatment and mobile health interventions in diabetes identify some effective interventions. There are a few trials testing the impact of brief messaging in type 2 diabetes, but they do not have systematically developed interventions and are at risk of bias. [4] Recent trials of Short Message Service (SMS) text-messaging for cardiovascular risk prevention and blood pressure lowering have shown clinically relevant changes in outcomes compared with usual care.[5]

Systematic reviews offer substantial evidence that tailored interventions are more effective than generic interventions. Tailored interventions may be seen by recipients as more personally relevant, so they will be more likely to attend to, read, understand, and act on them. In addition, tailored interventions are designed to change determinants of the target behaviour that are relevant to particular individuals (or to small subgroups of individuals); they therefore more precisely target the determinants of the individual's behaviour. There is scope for developing more highly tailored interventions for medication adherence using interactive voice response, SMS, and other digital platforms, although the potential benefit of increased effectiveness has to be balanced against the increased burden of measurement and greater complexity.

1.3 Preliminary data

In the formative work for this trial, we have examined which types of messages are most useful for people starting and taking a diabetes medicine and examined the extent to which messages can be tailored to the individual using routinely available NHS data. We have also explored the extent to which it is valuable for patients to decide on the types of messages to which they want to receive and respond.

1.4 Aims of current study

This trial is intended to inform the feasibility and design of a future clinical trial to estimate the extent of benefit and costs of a brief-messaging mobile based intervention compared to usual care in patients with type 2 diabetes as they start new medication and identify difficulties that might be encountered in practice. This research could offer a model for technology-based self-management support that could be extended to other aspects of diabetes care and other long-term conditions.

© Copyright: The University of Oxford and Oxford University Hospitals NHS Foundation Trust 2016

2 OBJECTIVES, STUDY PARAMETERS AND ENDPOINTS

2.1 Summary of objectives and outcomes

Objectives	Outcome Measures	Time-point(s) of evaluation of this outcome measure
To test participant recruitment to trial and willingness to be randomised.	Recruitment against planned recruitment rates.<br><br>Number of people showing an interest and not proceeding or those who withdraw from the control group and give a reason.	End of recruitment period.
i. To assess the feasibility of collection of clinical measurement data for the proposed 15-month clinical trial. (With particular interest in 15 months prior to trial entry or following trial entry measurement collection of participant's HbA1c, systolic blood pressure and cholesterol).<br><br>ii. To test willingness of participants to be followed up over the 26-week period post randomisation.<br><br>iii. To test the collection of prescribing data on trial participants.<br><br>iv. To test the collection of self-reported questionnaire data.<br><br>v. To assess the feasibility and acceptability of the intervention for patients and healthcare professionals (including general practitioners,	i. Completeness of data collection of HbA1c, systolic blood pressure and total to HDL cholesterol ratio.<br><br>ii. Retention and follow-up rates.<br><br>iii. Proportion of medication possession ratio for glucose, blood pressure and lipid lowering medication obtainable.<br><br>iv. Proportion of completed self-reported measures.<br><br>v. Data obtained through focus groups/qualitative interviews with patients and recruiting healthcare staff.	i. End of follow-up period.<br><br>ii. End of follow-up period.<br><br>iii. End of follow-up period.<br><br>iv. End of follow-up period.<br><br>v. Baseline and end of follow-up for patients and throughout the trial period for healthcare staff.

© Copyright: The University of Oxford and Oxford University Hospitals NHS Foundation Trust 2016

nurses, receptionists and pharmacists).<br><br>vi. To assess and understand the mechanisms of action of brief health related messages on self-reported adherence<br><br>viii. To assess feasibility and acceptability of self-completion of the resource use questionnaire.	vi. Change in quantitative process measures and relationship between changes in these measures and self-reported adherence.<br>vii. Proportion of completed resource use questionnaire.	vi. End of follow up period qualitative interviews/survey/telephone contacts with trial participants.<br><br>vii. End of follow-up period.
Exploratory measures	viii. Changes in primary and secondary measures that will be used in the main trial.<br><br>ix. Information on message delivery and interaction with participants.	viii. Differences between time points obtained from baseline to 26 weeks.<br><br>ix. Automated reports from messaging service on messages delivered and interactive messaging

2.2 Aims and objectives of the SuMMiT-D feasibility trial

The aim of this feasibility trial is to test recruitment of patients to, and collection of planned primary and secondary outcome data, for an effectiveness trial of a mobile phone based system intended to deliver brief, tailored behaviour change messages to people with type 2 diabetes focusing on use of medication. We will assess the feasibility and acceptability of the intervention for patients and healthcare professionals; the willingness of participants to be randomised; follow-up rates and trial procedures. We will also carry out a process evaluation of how the system is used and refine the way it is embedded within usual care.

2.2.1 Primary outcome

The primary outcome of this feasibility study is the rate of recruitment and randomisation of participants to the trial. We will measure recruitment against planned recruitment rates for the proposed main trial and number of people showing an interest in the trial and not proceeding or those who withdraw from the control group and give a reason.

2.2.2 Secondary outcomes

Secondary outcomes focus on feasibility of collection of clinical measurement data for the proposed main trial (where 15-month follow up is planned to ensure collection of clinical data collected annually). Of main interest is the feasibility of obtaining two measurements from the medical records, of HbA1c, systolic blood pressure and cholesterol from trial participants in a 15-month period, either prior to an entry measurement, or following trial entry. We will do this by collecting historical data to check frequency of HbA_{1c} measurement in the prospective trial population.

© Copyright: The University of Oxford and Oxford University Hospitals NHS Foundation Trust 2016
CONFIDENTIAL

Additional secondary outcomes are to measure follow-up of participants over a 26-week period and to test the collection of prescribing data on trial participants. We will obtain prescribing records for trial participants and report the proportion in which there is sufficient data to estimate availability of blood glucose, blood pressure and cholesterol lowering medications as a proportion of that expected. We will also measure the proportion of self-reported measures collected as the proportion of EQ-5D-5L. Potential mechanisms for the action of the brief messages will be assessed through changes in hypothesised health psychology constructs relating to use of medication, self-report medication adherence[6], and questions based on the Technology Acceptance model. [8] We will collect resource use data.

We will use qualitative methods to assess the feasibility and acceptability of the intervention for patients and also among health care workers (including general practitioners, nurses, receptionists and pharmacists).

3 TRIAL DESIGN

3.1 Intervention study

This is a primary care based, multicentre, 2-year (including a 26-week intervention), two-arm individually (1:1 ratio) randomised controlled, parallel group feasibility trial.

Patients with type 2 diabetes will be randomised to current care with individually tailored mobile device-based intervention encouraging and supporting them in developing a habit of taking their medication as intended and providing hints and tips to help them with other aspects of living with the condition (treatment arm) or to current care with the addition of infrequent non-health related messages (usual care arm).

The trial team will be blinded as to participant allocation to either the intervention or control arm. Investigators conducting qualitative interviews and focus groups with patients in the intervention arm will not be blinded to participant arm allocation but systems will be implemented to avoid clinicians or the trial team becoming aware of allocation status for individual participants. [*See Appendix A for study flowchart*].

Participants will receive messages for 26 weeks (approximately six months) from randomisation to final follow-up and remain in the trial for a further 18 months with data collected from their medical record. After the initial screening and eligibility check, informed consent will be obtained.

All participants will be followed-up at the end of the follow-up period post randomisation to collect questionnaire information (same as for baseline) and follow-up measures (same data collected for baseline). *[See Appendix B for a detailed schedule of procedures].*

3.1.1 Process evaluation (Cohort 1 - trial participants)

The process evaluation will consider mechanism, context and implementation using both quantitative and qualitative methods. The purpose of the evaluation will be to decide if any of the messages, or behaviour change techniques should be omitted prior to the main trial. Participants will be purposefully selected from the feasibility trial and invited to take part in qualitative interviews and post-follow up debriefing phone calls to share their views on the messaging system, insight into how it was implemented in daily life, identify issues around potential attrition and inform final procedures for the main trial (see *Section 4.1 Qualitative work with study participants* for further information). Up to 30 patients will be recruited.

If there is no change in the quantitative measure of proposed mechanisms, and there is qualitative evidence that certain behaviour change techniques (BCTs) and/or messages are disliked by participants, or have a negative impact on implementation these will be omitted prior to the main trial.

© Copyright: The University of Oxford and Oxford University Hospitals NHS Foundation Trust 2016

3.2 Interviews with Health Care Professionals from GP Practices (Cohort 2)

Finally, between 12 and 30 staff from GP practices taking part in the trial (the number based on considerations of data saturation) will also be invited to take part in focus groups and/or qualitative interviews to share their experience of recruiting for this trial, how the intervention was implemented in routine clinical care etc. (see *Section 4.2 Focus Group Work with Healthcare Professionals* for further information).

3.3 Treatment of Cohort 1 participants

3.3.1 INTERVENTION GROUP - Tailored mobile-phone messaging system

The intervention consists of brief, health related messages delivered via SMS text-message with content based on participant feedback received during formative work for this trial. The type of messages sent to participants are based on frequency of prescriptions issued to participants and other forms of real-time feedback from participants.

The intervention is a system with the following components (Appendix C provides a detailed description of the intervention components and their development).

i. Receipt of automated SMS text-messages relating to diabetes management and use of medicine.

ii. The library of messages includes different types of message using different behaviour change techniques (see appendix C for further details of the types of messages in relation to behaviour change techniques) to target health-related behaviour change relating to use of medicines, as well as messages targeting other aspects of diabetes care (e.g. diet and exercise).

iii. The types of messages sent (tailoring of messages) will be made more appropriate to individuals based on time since starting new medication and smoking status.

iv. Types and content of messages will also be linked to the records of prescribed medication details, appointment details and blood test results.

 Frequency of messages using a particular type of behaviour change technique will be modifiable based on interactive automated messaging about a participant's response to individual messages received.

v. Participants will be offered up to four text-messages per week with an average frequency of three per week.

vi. The style of messages will be patient-centred and will encourage patients to seek further associated information (including the use of links to selected external websites e.g. Diabetes UK).

The system for sending SMS text-messages is based on an NHS compliant computer network (see Appendix C).

3.3.2 CONTROL GROUP - Usual care

Participants assigned to usual care will be signed up to receive non-health related messages at a frequency of approximately one every four weeks. Care will otherwise not differ from usual care.

3.3.3 Potential risks and benefits of the tailored mobile-phone messaging system

We do not anticipate harms from using this system. The messages are carefully designed to avoid requiring specific changes in management, rather they are aimed at supporting behaviours

© Copyright: The University of Oxford and Oxford University Hospitals NHS Foundation Trust 2016

around use and maintenance of recommended treatments. We will make standard recommendations about safety in use of mobile phones and devices in the PIS.

Participants will be able to interact with the system. This will give them the opportunity to influence the types and frequencies of the intervention messages to their individual needs and preferences. They will also be given the option to pause and restart the messages and will be able to contact the trial team at any time if they would like to stop receiving messages or withdraw from the trial.

The major benefit of the intervention would be the information and advice participants receive about their condition. This may increase their understanding of type 2 diabetes and provide support with managing their condition.

3.4 PARTICIPANT IDENTIFICATION

3.4.1 Trial Participants – Cohort 1

Participants are people with type 2 diabetes registered with a UK general practitioner (GP). Eligible patients should meet all of the inclusion and have none of the exclusion criteria listed below. Any queries regarding patient eligibility should be directed to the trial team prior to randomisation. No more than one individual within the same household will be recruited and participants will be asked not to share messages.

3.4.1.1 Inclusion Criteria

- Participant is willing and able to give informed consent for participation in the trial.
- Male or Female, ≥35 years of age.
- Type 2 diabetes.
- Taking oral glucose lowering treatment, blood pressure lowering treatment or lipid lowering treatment either alone or in combination.
- Has started one or more of these medications or had a change in one of these medications within the last three months.
- Has access to a mobile phone and is able, if necessary with help (e.g. relative, friend, neighbour), to send, understand and retrieve brief SMS text-messages in the English language.
- The participant's practice is participating in the trial.

3.4.1.2 Exclusion Criteria

The participant may not enter the trial if ANY of the following apply:

- Female participant who is pregnant, within three months post-partum or planning pregnancy during the course of the trial.
- A serious medical condition that, in the opinion of the investigator, makes them ineligible.
- Insulin treatment without also concomitant use of oral glucose lowering treatment.
- Patient has been admitted to hospital within the last three months for hyper- or hypoglycaemia (self-report)
- Another person in the household already participates in this trial

3.4.2 Staff from GP Practices – Cohort 2

- Inclusion criteria – staff are aware of the text-messaging intervention and have contact with patients or the management of patients with type 2 diabetes (see *Section 4.2 Focus Group Work with Healthcare Professionals* for further information).

© Copyright: The University of Oxford and Oxford University Hospitals NHS Foundation Trust 2016

- Participant is willing and able to give informed consent for participation in the study.

- No exclusions.

3.5 TRIAL PROCEDURES

See Appendix B for a summary of the trial procedures.

3.5.1 Recruitment procedures - Cohort 1

Potential participants will be identified primarily through general practices across the UK. Alternative routes to approach potential participants in areas to support practices in recruiting patients e.g. condition specific mailing lists, databases of 'research-interested' people, pharmacies, community centres, libraries, other public areas, social media, recruitment advertisements in condition-specific charities, local news outlets, GP practice websites, GP practice waiting areas, trial website etc. will be considered and used if necessary to explore alternative modes of engaging people and encouraging them to consider checking whether their practice is taking part in the trial. To raise awareness about the trial and opportunities for participation, a short information leaflet may be provided to patients via a variety of methods including post, e-mail, displayed at sites' waiting areas, given to patients by a practice team member and the alternative routes described above.

Healthcare professionals will screen their type 2 diabetes clinic lists for potentially eligible patients and also identify participants attending opportunistically. Where possible, health care professionals may be asked to screen their clinic lists and mail-out to potentially eligible patients. Following an initial mail-out, subsequent searches may be carried out to identify newly eligible patients. Newly eligible participants may also be contacted by a number of methods (including phone, letter, email or text-message), by the practice team up to three times.

People who may be interested in taking part in the trial will be asked to send their name by SMS text-message to the trial team to express this interest. If potential participants have any difficulties in registering their interest in the trial in this way, they will be able to call a Freephone trial telephone number or email the trial team and will receive support in registering as required. Following their verbal consent, people who contacted the trial team will be given the opportunity to discuss the trial further by telephone and, if there is interest and the potential participant is willing, carry out a screening interview to collect data about eligibility.

Recruitment of Healthcare Professionals (Cohort 2)

See section 4.2 for information on identification and consent of healthcare professionals.

3.5.2 Screening Assessment

Following a participant's expression of interest (via text, phone or email), provision of further information about the study and screening for eligibility, may take place within the same phone call or a subsequent contact with the patient may be arranged, if needed, to answer any questions they may have.

Participants will be able to ask their healthcare professional or the members of the trial team any questions they have and the team will confirm if they are eligible to take part in the trial prior to completing informed consent.

Eligible patients will need to meet all the inclusion criteria and not meet any of the exclusion criteria described in sections 3.4.1.1 and 3.4.1.2.

Participants that have been found ineligible due to their GP practice not being a recruiting practice may be screened again, above provision of verbal consent to be approached if their practice signs up to the trial during the recruitment period.

© Copyright: The University of Oxford and Oxford University Hospitals NHS Foundation Trust 2016

3.6 Informed Consent

Participants will be provided with the participant information leaflet and have to sign a consent form prior to any trial procedures being performed. Participants will personally sign, write their full name and date the consent form, before any trial specific procedures are performed (or complete with their full name and personal password (also referred to as access code), if done electronically).

Participants will be given as much time as they wish to consider the trial information (at least 48 hours before any subsequent contacts are made to confirm receipt of the forms and answer any questions the participant may have).

Participants will be advised that they may be contacted throughout the 26-week follow-up period should any issues arise (e.g. the participant is not able to receive messages), and that the last contact with the team will be at 26 weeks (± 4 weeks) to collect information from the follow-up questionnaires. They will be advised that we will ask for information from their medical records about medication, blood tests and other related health data for up to two years. Participants will be informed that if they agree they may be invited to participate in a qualitative sub-study during which additional contacts may be made (see section 4.1).

It will be clearly stated in the participant information leaflet that the participant is free to withdraw from the trial at any time for any reason without that affecting their care or legal rights and with no obligation to give the reason for withdrawal. However, as one of the trial objectives is to test “participants’ willingness to be randomised”, those who withdraw will be asked a question whether they decided to withdraw because they were not randomised to the intervention group.

If completing consent online, the participant will be asked to enter their full name, and their password (provided by the trial team) to verify their identity and access the participant information leaflet and e-consent form. Informed consent for those completing paper-based forms will be obtained by means of a participant dated signature.

The participant will be required to agree to each statement on the electronic consent form in the same way that they would complete the paper based consent form, and write their full name and date before submitting it. The combination of personal password, full name and date will form the electronic signature for each person participating in this trial.

A copy of the signed informed consent will be provided to the participant via email or post according to the Nuffield Department of Primary Care Health Science’s Information Governance toolkit. . A second copy will be provided to the participant’s GP to be kept with their patient’s medical records.

Due to the low risk posed by the nature of the trial, participants may be consented and have their baseline assessments completed on the same day as the day of expression of interest. Participants can subsequently withdraw consent at any point in the trial without penalty having taken more time to consider participation.

3.7 Randomisation, blinding and code-breaking

A secure web-based randomisation system will be provided by the Oxford Primary Care Clinical Trials Unit (PC-CTU) using the Sortition software programme. Participants will be allocated to one of the two arms: Intervention Group - usual care with the support of a mobile based intervention or Control Group - usual care with the addition of non-health related messages on a 1:1 basis. Allocation will be carried out using a non-deterministic minimisation algorithm to ensure intervention groups are balanced for important baseline prognostic and other factors: study site, age <65/≥65 years), gender (M/F), duration of diabetes (<5 years/≥5 years), number of medications (<5/≥5).

If the web-based randomisation system is not accessible, allocation will be carried out following emergency randomisation procedures outlined in the trial working instructions.

© Copyright: The University of Oxford and Oxford University Hospitals NHS Foundation Trust 2016

Participant allocation will not be formally blinded to healthcare professionals but to avoid contamination around outcome measurement no records of allocation outwith the trial database will be made. As this is a low risk feasibility trial, unblinding procedures are unlikely to be needed. However, in the event of a participant's clinical condition necessitating breaking the code, this will be done according to the relevant PC-CTU SOP or trial specific working instruction and the group allocation will be sent to the chief investigator, site principal investigator or patient's GP.

3.8 Baseline Assessment

Participants will be invited to access the participant information leaflet, consent form and baseline questionnaires on the trial website. Each participant will be given a unique personal password to allow access to the trial website. A unique personal password in combination with the volunteer's full name will form part of their electronic signature that will enable them to log in to the trial website and complete their baseline forms (including participant information leaflet, consent form and baseline questionnaires).

Alternatively and on request, participant information leaflet, consent form and baseline questionnaires will be sent by post to potential participants. Members of the trial team will be available to offer support, guidance in using the online system and further information at the same time as completing the forms, if needed.

A Freephone number to contact the trial team will be made available. The participant will be able to contact the trial team whenever needed to ask any questions they have, including questions they have prior to completing their consent form.

3.8.1 Baseline assessment procedures

After eligibility is confirmed and consent has been taken, the following procedures will take place:

- Contact details will be collected on the phone, or online and will be updated where there is information missing from previous contacts.
- Participant completion of baseline questionnaires. These are:
  - MARS self-report scale[6] (Questions about using your medicines)
  - EQ-5D-5L[7] (Health status EQ-5D-5L)
  - A measure based on health psychology theory and the technology acceptance model. (Ideas and concerns about your diabetes treatment)
  - Healthcare utilisation record (Health services use)
  - Specific questions about experience of diabetes education, presence of a carer and their role in dose administration, duration of diabetes, if the pharmacy used by the participant automatically requests patient's medication from surgery, self-reported level of education, smoking, age, gender, ethnicity, NHS number, Date of Birth, familiarity with mobile phones and computers, and details of current mobile phone including contract.

  All self-report data will be collected either on paper forms or electronically.

  Participants will be contacted at least 48 hours after they have been provided with their baseline forms to confirm they have received them.
  If the forms have not been submitted or received by the trial team 7-14 days from the date they were sent by the team, further contacts will be made.

- The following baseline measures will be obtained from the participant's medical record and provided to the trial team. For the period of 15 months prior to recruitment: HbA1c,

© Copyright: The University of Oxford and Oxford University Hospitals NHS Foundation Trust 2016

systolic and diastolic blood pressure, total and HDL cholesterol, weight, height, current medication and prescriptions for diabetes drugs (glucose lowering, blood pressure lowering and lipid lowering. Previous occurrence of myocardial infarction, stroke, Transient Ischemic Attack (TIA), heart failure, peripheral vascular disease and renal failure. Data collection will be through one of the following routes: provision of a medical summary by the GP practice team, or a baseline CRF completed by a nurse or facilitator or a trial team member visiting the surgery.

3.8.2 Follow-up (data collection)

Follow-up will last for 26 weeks after randomisation. For ease of reference, all patient facing documentation will refer to this period as 'six months'. To address any issues that may arise (e.g. the participant is not able to receive messages), an unscheduled contact may be made during the 26 week follow up period if required.

Participants will be asked to complete the following questionnaires at 26 weeks (± 4 weeks) following randomisation:

- MARS self-report scale[6]
- EQ-5D-5L[7]
- A measure based on health psychology theory and the technology acceptance model.[8]
- Healthcare utilisation record

The 26-week follow-up questionnaires will be posted to the participant or a link will be given to them via email. If the participant's follow-up questionnaires have not been received one week after the due date, the trial team will contact the participant to remind them to complete these online or on paper. Multiple attempts will be made within the determined window (= ± 4 weeks).

If participants choose to complete the 26-week measures online, they will be required to log in to the electronic questionnaires using the same electronic verification process used at baseline assessment.

Follow up data will be obtained from the medical record and provided to the trial team for the period of 26 weeks after recruitment: HbA1c, systolic and diastolic blood pressure, total and HDL cholesterol, weight, height, current medication and prescriptions for diabetes drugs (glucose lowering, blood pressure lowering and lipid lowering). Data collection will be through one of the following routes: provision of a medical summary provided by the GP practice team or a 26-week notes review CRF completed by a nurse or facilitator or a trial team member visiting the surgery.

3.9 Long term follow up

We will obtain consent to collect routine medical data from participants' GP notes two years after randomisation. This will allow exploratory data to be obtained about longer term outcomes to inform the analysis of data from future studies.

3.10 Discontinuation or withdrawal of participants from trial

Each participant has the right to withdraw from the trial at any time. In addition, the Investigator may discontinue a participant from the trial at any time if the Investigator considers it necessary for any reason including:

- Pregnancy
- Ineligibility (either arising during the trial or retrospectively having been overlooked at screening)
- Significant protocol deviation

© Copyright: The University of Oxford and Oxford University Hospitals NHS Foundation Trust 2016

- Significant non-compliance with trial requirements
- Disease progression which renders further data collection inappropriate
- Withdrawal of consent for data collection
- Loss to follow up

An intention-to-treat analysis will be adopted, so if a participant withdraws from the trial, their data up to that point will be used in analysis.

The reason for withdrawal will be recorded in the relevant CRF as per the PC-CTU SOP

Withdrawn participants will not be replaced.

Participants may decide to stop receiving text messages. **This does not constitute withdrawal from the trial.** Data collection will continue unless declined.

3.11 Definition of End of Trial

The end of trial is the date of the last data capture.

4 Process analysis

4.1 Qualitative work with Cohort 1 study participants

We are aiming to recruit up to 30 patients with type 2 diabetes from the intervention group only. The trial participant information leaflet will include information about the nature of the qualitative study and what it will involve for the participant if they decide to take part.

Participants in the interview study will be informed that their participation is entirely voluntary and if they do not wish to take part this will not affect their involvement in the feasibility trial or their care in any way.

Participants who have given consent will be interviewed twice. At the initial interview, participants will be asked questions including, sharing their experience of managing their diabetes medications, challenges with managing their condition etc.
At the final interview, participants will be asked to share their views on the system and help the investigators understand how the messages were perceived, how they influenced adherence and self-management in general and identify issues around potential attrition and inform the final design of the main trial.

We will use purposeful sampling to recruit a diverse group of participants in terms of age, gender, duration of diabetes, ethnicity, socioeconomic status, comorbidity, medication use, illness experiences and familiarity with technology.

Interviews will be conducted over the phone at the beginning and the end of the 26-week follow up period (± 4 weeks).They will be audio recorded and transcribed verbatim (the transcripts will be anonymised and will be kept in a secure location to protect the participants' anonymity and confidentiality).

4.2 Focus Group Work with Cohort 2 Health Care Professionals

We are aiming to recruit a minimum of 12 and up to 30 staff at GP practices that are taking part in the trial to take part in focus groups or individual interviews. The final sample size will be determined by data saturation that is when no novel categories, themes or explanations are identified in additional interviews [9].

Each focus group will take place on one occasion. Additional face-to-face, telephone interviews or surveys will also be conducted if key staff are unavailable to attend planned focus groups.

© Copyright: The University of Oxford and Oxford University Hospitals NHS Foundation Trust 2016

Healthcare professionals will be informed that we will be recruiting for this qualitative study at the site initiation visit/study training. They may then be approached with information about the study, mainly via email or phone.

Healthcare professionals that express interest in taking part will be provided a participant information leaflet and consent form via email or post. The participant information leaflet will detail the nature of the study, what it will involve for the participant and any risks involved in taking part. Participation is entirely voluntary.

Where healthcare professionals are interviewed face-to-face a consent form will be provided and signed before the interview commences. Where interviews are carried out over the phone, consent will be audio recorded. The researcher will read each of the statements from the consent form and ask the participant if they agree; this will be audio recorded to document their informed consent.

The main purpose of this sub-study will be to identify the context of, and potential barriers and facilitators to implementation of the text-based system as a tool providing support for adherence to medications for patients with type 2 diabetes who have had a recent change in medication or were recently prescribed a new treatment. Specifically, we will explore staff experiences of recruiting for this study, how the intervention was implemented during the feasibility trial, and how the intervention could be implemented into routine clinical care.

Data collection will run throughout the trial period defined in this protocol.

Focus group discussions and interviews will be audio recorded and transcribed verbatim. The transcripts will be anonymised and kept in a secure location with access to authorised personnel only to protect participants' anonymity and confidentiality.

If a participant withdraws from the study, their data up to that point will be used in the analysis. Especially, for participants taking part in focus groups, it will not be made possible to isolate and remove individual comments from a group discussion.

5 SAFETY REPORTING

5.1 Definitions

An elective hospital admission will not be considered a serious adverse event.

Adverse Event (AE)	Untoward medical occurrences which are not necessarily caused by or related to the intervention will not be collected as part of the feasibility trial.
Adverse Reaction (AR)	An untoward and unintended response in a participant which is related to a participant using their mobile phone as part of this intervention.<br><br>"Response" means that a causal relationship between using a mobile phone as part of this intervention (i.e. to read messages) and an AE is at least a reasonable possibility, i.e. the relationship cannot be ruled out.<br><br>All cases judged by either the reporting medically qualified professional or the Sponsor as having a reasonable suspected causal relationship to the intervention qualify as adverse reactions.

© Copyright: The University of Oxford and Oxford University Hospitals NHS Foundation Trust 2016

Serious Adverse Event (SAE)	A serious adverse event is any untoward medical occurrence that:<br>• results in death<br>• is life-threatening<br>• requires inpatient hospitalisation or prolongation of existing hospitalisation<br>• results in persistent or significant disability/incapacity<br>• consists of a congenital anomaly or birth defect.<br>Other 'important medical events' may also be considered serious if they jeopardise the participant or require an intervention to prevent one of the above consequences.<br>N.B. The term "life-threatening" in the definition of "serious" refers to an event in which the participant was at risk of death at the time of the event; it does not refer to an event that hypothetically might have caused death if it were more severe.<br>N.B. In this trial, we will <u>not</u> be collecting SAEs for:<br>○ scheduled hospital visits<br>○ hospitalisation due to diabetes or any other underlying conditions<br>○ congenital anomalies or birth defects<br>○ any life-threatening medical occurrence that is not related to the intervention or participation in the trial
Serious Adverse Reaction (SAR)	An adverse event that is both serious and, in the opinion of the reporting Investigator, believed with reasonable probability to be due to one of the trial treatments, based on the information provided e.g. a car accident due to mobile phone use while driving resulting in inpatient hospitalisation.
Suspected Unexpected Serious Adverse Reaction (SUSAR)	A serious adverse reaction, the nature and severity of which is not consistent with the information about the intervention in question.

Other 'important medical events' may also be considered serious if they jeopardise the participant or require an intervention to prevent one of the above consequences.

The term "life-threatening" in the definition of "serious" refers to an event in which the participant was at risk of death at the time of the event; it does not refer to an event which hypothetically might have caused death if it were more severe.

To avoid confusion or misunderstanding of the difference between the terms "serious" and "severe": "Severe" is often used to describe intensity of a specific event, which may be of relatively minor medical significance. "Seriousness" is the regulatory definition supplied above.

5.2 Causality

The relationship of each SAE to the trial intervention must be determined by a medically qualified individual according to the following definitions:

Unrelated: Where an event is not considered to be related to the intervention. The adverse event is probably produced by the participant's clinical state or by other modes of therapy administered to the participant.

© Copyright: The University of Oxford and Oxford University Hospitals NHS Foundation Trust 2016

Possibly related: Although a relationship to the intervention cannot be completely ruled out, the nature of the event, the underlying disease, concomitant medication or temporal relationship make other explanations possible.

Probably related: The temporal relationship and absence of a more likely explanation suggest the event could be related to the intervention.

Definitely related: The known effects of the intervention, its therapeutic class or based on challenge testing suggest that the intervention is the most likely cause.

All SAEs labelled possibly, probably or definitely related will be considered as related to the intervention.

5.3 Procedures for Recording Adverse Events

We do not anticipate any AEs to result as part of the intervention and as a result AEs will not be recorded.

5.4 Reporting Procedures for Serious Adverse Events

All SAEs that occur from randomisation until the end of the participant's 26-week follow up period, either observed by the recruiting clinician or reported by the participant, will be recorded and forwarded to PC-CTU, using the PC-CTU SAE Report form following assessment for seriousness and relatedness by the site clinician. This form will be completed and sent to PC-CTU using secure email or other secure methods as outlined on the report form. As a minimum, the following information will be recorded:

- Description
- Date of onset
- End date
- Severity
- Assessment of relatedness to study intervention
- Other suspect drug or device
- Action taken

Follow up information should be provided as necessary. SAEs must be reported to the PC-CTU within 24 hours of discovery or notification of the event. PC-CTU will be acting on behalf of the sponsor that is Oxford University.

The PC-CTU will acknowledge receipt of the SAE Report Form using the relevant PC-CTU documentation. This receipt will be emailed and faxed to the site clinician. If the site clinician does not receive a receipt within 24hrs of them sending the report (during office hours), they should re-send the SAE Report Form to the PC-CTU by email or fax and telephone ahead.

The documentation will be reviewed by members of the Trial Management Group and the 'SAE Checklist' will be completed and retained by the PC-CTU. Following the initial check of the report, any additional information will be requested, and the CI or their medically qualified designated representative will review and evaluate the report for seriousness, causality and expectedness. The Data Monitoring Committee (DMC) will agree prospectively how SAE reports will be reviewed.

Additional information, as it becomes available, will also be reported on the paper SAE Report Form (i.e. updating the original form) and returned to the PC-CTU. The SAE Report Form will be filed in the Trial Master File according to the relevant PC-CTU Standard Operating Procedure (SOP), with copies filed in the patient's medical notes and the Investigator Site File (if the participant is recruited through a Primary Care recruiting site).

SAEs will be monitored by the trial manager and any concerns identified will be immediately raised with the CI and may be tabled for discussion at the regular PC-CTU Management Committee meetings or referred to the trial's DMC for review. The DMC also monitors the

© Copyright: The University of Oxford and Oxford University Hospitals NHS Foundation Trust 2016

frequency and pattern of events reported as part of its independent oversight of the trial. Reporting will only include SAEs that are attributed to trial intervention or participation in the trial, **Foreseeable and predefined SAEs, e.g. if hospital admission is expected, do not need to be reported. Additionally, we will not be recording SAEs that are related to diabetes or any other underlying medical conditions** (See section 12.1 above).

6 STATISTICS

6.1 Description of Statistical Methods

The analysis will be carried out once the final patient has completed their 26-week follow up. An intention to treat approach will be used, where all participants randomised will be included in the group to which they were allocated, regardless of intervention received. The results of the primary and secondary objectives will be presented descriptively using proportions and 95% confidence intervals.

The primary outcome will be the number of patients recruited and randomised as a proportion (with 95% confidence interval) of the target recruitment number.

Secondary outcomes, including loss to follow up (%), withdrawals (%) and completeness of data collection for various outcomes (%), will be presented overall and separately by treatment group.

Exploratory outcomes (changes in primary and secondary measures to be used in the main trial) will be analysed using an intention to treat approach. Continuous outcomes will be analysed using an analysis of covariance adjusting for minimisation factors. Results will be presented as a difference in means with 95% confidence interval. Binary outcomes will be analysed in a similar way using log binomial regression models (adjusting for minimisation factors). Results will be presented as relative risks with 95% confidence intervals.

A detailed statistical analysis plan will be prepared prior to data lock.

6.2 The Number of Cohort 1 Participants

With 200 participants (100 in each group) the feasibility trial is powered to detect 80% follow up within 95% confidence intervals of 73.8% to 85.3%.

6.3 The number of Cohort 2 Participants

No formal sample size calculation is required for this Cohort. We aim to recruit between 12 and 30 healthcare professionals based on considerations of data saturation.

7 DATA MANAGEMENT

7.1 Source Data

Source documents are where data are first recorded, and from which participants' CRF (paper or electronic) data are obtained. These include, but are not limited to, hospital records (from which medical history and previous and concurrent medication may be summarised into the CRF), clinical and office charts, laboratory and pharmacy records, diaries, microfiches, radiographs, and correspondence.

CRF entries will be considered source data if the CRF is the site of the original recording (e.g. there is no other written or electronic record of data). All documents will be stored safely in confidential conditions. On all trial-specific documents, other than the signed consent, the participant will be referred to by the trial participant number/code, not by name.

© Copyright: The University of Oxford and Oxford University Hospitals NHS Foundation Trust 2016

8 Access to Data

Direct access will be granted to authorised representatives from the Sponsor, host institution (University of Oxford) and the regulatory authorities to permit trial-related monitoring, audits and inspections. To ensure data transparency, the trial will be registered on the International Standard Randomised Controlled Trial Number (ISRCTN) registry before the first participant is recruited.

8.1 Data Recording and Record Keeping

All trial data where feasible will be entered onto electronic CRFs directly on the trial clinical database. This clinical database will be built and managed by the PC-CTU in line with the PC-CTU SOPs and will hold and allow data management of all data points required to conduct the final analysis. The clinical database will be built on an externally validated secure web-based platform allowing for data tracking by use of date stamped audit logs. In this database, participants will be identified only by a unique trial number to protect from bias and ensure the participant's confidentiality.

Participant identifiable information will be stored in a separate encrypted, secure database, only accessible by trial team members with member-specific log-ins. Participant contact information will be deleted and securely purged from the system six to twelve months after the study has finished.

Paper CRFs will be completed if needed, i.e. if the system is updated and is temporarily unavailable or a participant have opted to complete the data requested on paper. The participants will be identified by a unique trial specific number. The name and any other identifying details will NOT be included in any trial data electronic file or paper CRF.

The study protocol, documentation, data and all other information generated will be held in strict confidence. No information concerning the study, or the data will be released to any unauthorised third party, without prior written approval of the sponsor. A clinical data manager will be assigned to the trial supervised by senior members of the Oxford PC-CTU and PC-CTU SOPs will be followed.

The intervention tested in this trial includes linkage of limited data (medications, appointments, blood test results) from the GP clinical systems to an NHS based computer for the purpose of delivering reminders and prompts to patients when medication may not have been prescribed as planned. This data will be transferred from GP computer systems and securely stored in an NHS compliant computer system. Agreements will be in place between the data supplier, the data processor and individual practices (data owner) setting out responsibilities for the secure handling of this data. This data will only be accessed by members of the research team holding honorary NHS contracts for the purposes of system maintenance. This data will not be accessed, used or stored for secondary use.

All interviews will be recorded using a digital recorder and participants will be asked to consent to this as part of the informed consent process. Written notes will not be made during the interview. Interviews will be uploaded from the digital recorder following the interview, checked and deleted from the recording device. Audio data will be saved on a password-protected university network computer. Interviews will be encrypted before being sent for transcription using the University's secure file-transfer service. Upon receipt, transcripts will be checked for accuracy and anonymised. Transcripts will be password protected and saved on a password-protected university network computer.

9 QUALITY ASSURANCE PROCEDURES

The trial will be conducted in accordance with the current approved protocol, Good Clinical Practice (GCP), relevant regulations and PC-CTU standard operating procedures.

© Copyright: The University of Oxford and Oxford University Hospitals NHS Foundation Trust 2016

Regular monitoring will be performed according to GCP. Data will be evaluated for compliance with the protocol and accuracy in relation to source documents. Following written standard operating procedures, the monitors will verify that the trial is conducted, and data are generated, documented and reported in compliance with the protocol, GCP and the applicable regulatory requirements.

9.1 Trial Management Group

The Trial Management Group (TMG) will be responsible for the monitoring of all aspects of the trial's conduct and progress and will ensure that the protocol is adhered to and that appropriate action is taken to safeguard participants and the quality of the trial itself. The TMG will be comprised of individuals responsible for the trial's day-to-day management (e.g. the CI, trial manager, statistician, data manager) and will meet regularly throughout the course of the trial.

9.2 Trial Steering and Data Monitoring Committees

This trial is a feasibility study and as such will not have a Trial Steering Committee (TSC) or Data Monitoring Committee (DMC). In preparation for a full-trial an independent Trial Steering Committee will be convened to review progress and provide advice to the investigators about all aspects of the future trial.

9.3 Trial Coordinating Centre

This is a multicentre trial, comprised of two recruiting centres: lead centre and local coordinating centre. The lead recruiting centre will ensure that the Sponsor's responsibilities are carried out in their recruiting region. The responsibilities of the lead recruiting centre for activities, including ensuring study materials are available, GP practice staff is appropriately trained etc., may be delegated to the local coordinating centre for practices in their recruiting region.

10 ETHICAL AND REGULATORY CONSIDERATIONS

10.1 Declaration of Helsinki

The Investigator will ensure that this trial is conducted in accordance with the principles of the Declaration of Helsinki.

10.2 Guidelines for Good Clinical Practice

The Investigator will ensure that this trial is conducted in accordance with relevant regulations and with the Good Clinical Practice.

10.3 Approvals

The protocol, informed consent form, participant information leaflet and any proposed advertising material will be submitted to an appropriate Research Ethics Committee (REC), Health Research Authority (HRA), regulatory authorities and host institution for written approval.

The Investigators will submit and, where necessary, obtain approval from the above parties for all substantial amendments to the original approved documents.

10.4 Reporting

The CI will submit once a year throughout the clinical trial, or on request, an Annual Progress Report to the REC, host organisation, funder and Sponsor. In addition, an End of Trial notification and final report will be submitted to the REC, host organisation and Sponsor.

© Copyright: The University of Oxford and Oxford University Hospitals NHS Foundation Trust 2016

10.5 Participant Confidentiality

The trial staff will ensure that the participants' anonymity is maintained. Interview transcripts will be anonymised. The participants will be identified only by a unique participant ID number on all trial documents and any electronic database.

Participants' contact details needed to contact participants on an ongoing basis, e.g. for follow-up, and any other participant identifiable information (e.g. NHS number), will be held securely and separately from the anonymised data. All documents will be stored securely and will only be accessible by trial staff and authorised personnel. The trial will comply with the Data Protection Act, which requires data to be anonymised as soon as it is practical to do so.

10.6 Expenses and Benefits

Upon return of their 26-Week Follow up Questionnaires, participants will be sent a £10 voucher. Reasonable travel expenses will be reimbursed to focus group participants for travelling to/from the focus group meeting venue. Reimbursement will be made on production of receipts, or a mileage allowance provided.

10.7 Other Ethical Considerations

We do not anticipate any other ethical considerations not previously identified and addressed in this document.

11 FINANCE AND INSURANCE

11.1 Funding

This study is funded by the NIHR Programme Grants for Applied Research as part of a wider programme of work (RP-PG-1214-20003).

11.2 Insurance

The University has a specialist insurance policy in place that would operate in the event of any participant suffering harm as a result of their involvement in the research (Newline Underwriting Management Ltd, at Lloyd's of London).

12 PUBLICATION POLICY

The investigators will be involved in reviewing drafts of the manuscripts, abstracts, press releases and any other publications arising from the study. Authors will acknowledge that the study was funded by NIHR Programme Grants for Applied Health Research, give the appropriate 28-day notice and carry the standard disclaimer. Authorship will be determined in accordance with the ICMJE guidelines and other contributors will be acknowledged.

This trial may lead to the development of new IP. Ownership of IP generated by employees of the University vests in the University. The protection and exploitation of any new IP is managed by the University's technology transfer office, Oxford University Innovations.

© Copyright: The University of Oxford and Oxford University Hospitals NHS Foundation Trust 2016

13 REFERENCES

1. Ogurtsova, K. *et al.* IDF Diabetes Atlas: Global estimates for the prevalence of diabetes for 2015 and 2040. *Diabetes Res Clin Prac* **128,** 40–50 (2017).
2. Salas, M., Hughes, D., Zuluaga, A., Vardeva, K. & Lebmeier, M. Costs of Medication Nonadherence in Patients with Diabetes Mellitus: A Systematic Review and Critical Analysis of the Literature. *Value Health.* **12,** 915–922 (2009).
3. Haynes RB, A. E. S. N. M. H. Y. X. Interventions for enhancing medication adherence (Review). 1–160 (2012).
4. Farmer, A. J. *et al.* Effects of interventions promoting monitoring of medication use and brief messaging on medication adherence for people with Type 2 diabetes: a systematic review of randomized trials. *Diabet Med* **33,** 565–579 (2015).
5. Bobrow, K. *et al.* Mobile Phone Text Messages to Support Treatment Adherence in Adults With High Blood Pressure (SMS-Text Adherence Support [StAR]): A Single-Blind, Randomized Trial. *Circulation* **133,** 592–600 (2016).
6. Horne, R. & Weinman, J. Patients' beliefs about prescribed medicines and their role in adherence to treatment in chronic physical illness. *J.Psychosom.Res.* **47,** 555–567 (1999).
7. Herdman, M. *et al.* Development and preliminary testing of the new five-level version of EQ-5D (EQ-5D-5L). *Qual Life Res* **20,** 1727–1736 (2011).
8. Venkatesh, V. & Davis, F. D. A Theoretical Extension of the Technology Acceptance Model: Four Longitudinal Field Studies. *Management Science* **46,** 186–204 (2000).
9. Marshall, M. N. Sampling for qualitative research. *Fam.Pract.* **13,** 522–525 (1996).
10. Schwarzer, R. **Modeling Health Behavior Change: How to Predict and Modify the Adoption and Maintenance of Health Behaviors**. *Applied Psychology: an International Review* **57,** 1–29. (2008)

© Copyright: The University of Oxford and Oxford University Hospitals NHS Foundation Trust 2016

14 APPENDIX A: TRIAL FLOW CHART – Cohort 1

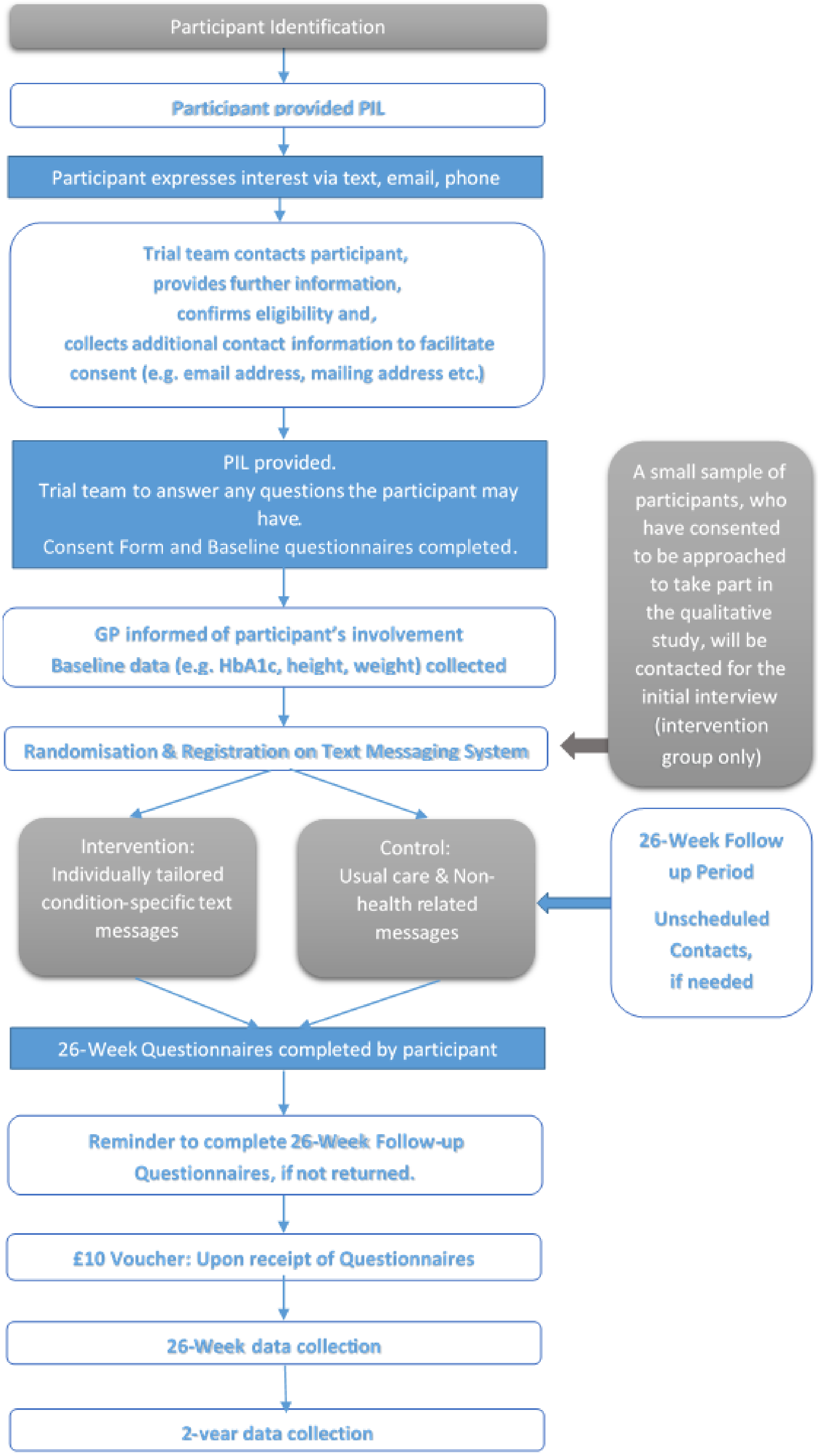

© Copyright: The University of Oxford and Oxford University Hospitals NHS Foundation Trust 2016

15 APPENDIX B: SCHEDULE OF PROCEDURES TABLE & TIMELINE – Cohort 1

	Contact Timing				
	Recruitment Method Dependent, e.g. at GP appointment, prior to mail-out GP screening their type 2 diabetes clinic list etc.	Day participant expression of interest received – Or as soon as possible thereafter.	0-26 Weeks (Randomisation is day 1 of Week 0)	26 Weeks (± 4 weeks)	Year-Two (From Randomisation)
**Cohort 1 – Trial Participants**					
Screening	x				
Eligibility		x			
Informed Consent		x			
Demographics and additional information Questionnaire		x			
MARS self-report scale - Questionnaire		x		x	
EQ-5D-5L Health status - Questionnaire		x		x	
Healthcare Utilisation Record Questionnaire		x		x	
Health Psychology & Technology Acceptance Questionnaire		x		x	
Baseline data collection (including medical history and concomitant medication)		x			
Randomisation		x			
Text Messaging System Registration		x			
Intervention or Control procedures			x		
Scheduled and Unscheduled Contacts	x	x	x	x	
26-Week (± 4 weeks) Data collection				x	
Qualitative patient interviews for intervention group			x	x	
Year-two data collection from practices					x

© Copyright: The University of Oxford and Oxford University Hospitals NHS Foundation Trust 2016

Cohort 2: Eligibility, informed consent and group or individual interviews or surveys will be taking place with healthcare professionals (whose practices are taking part in the feasibility) throughout the trial period.

© Copyright: The University of Oxford and Oxford University Hospitals NHS Foundation Trust 2016

15 APPENDIX B: Trial Timeline

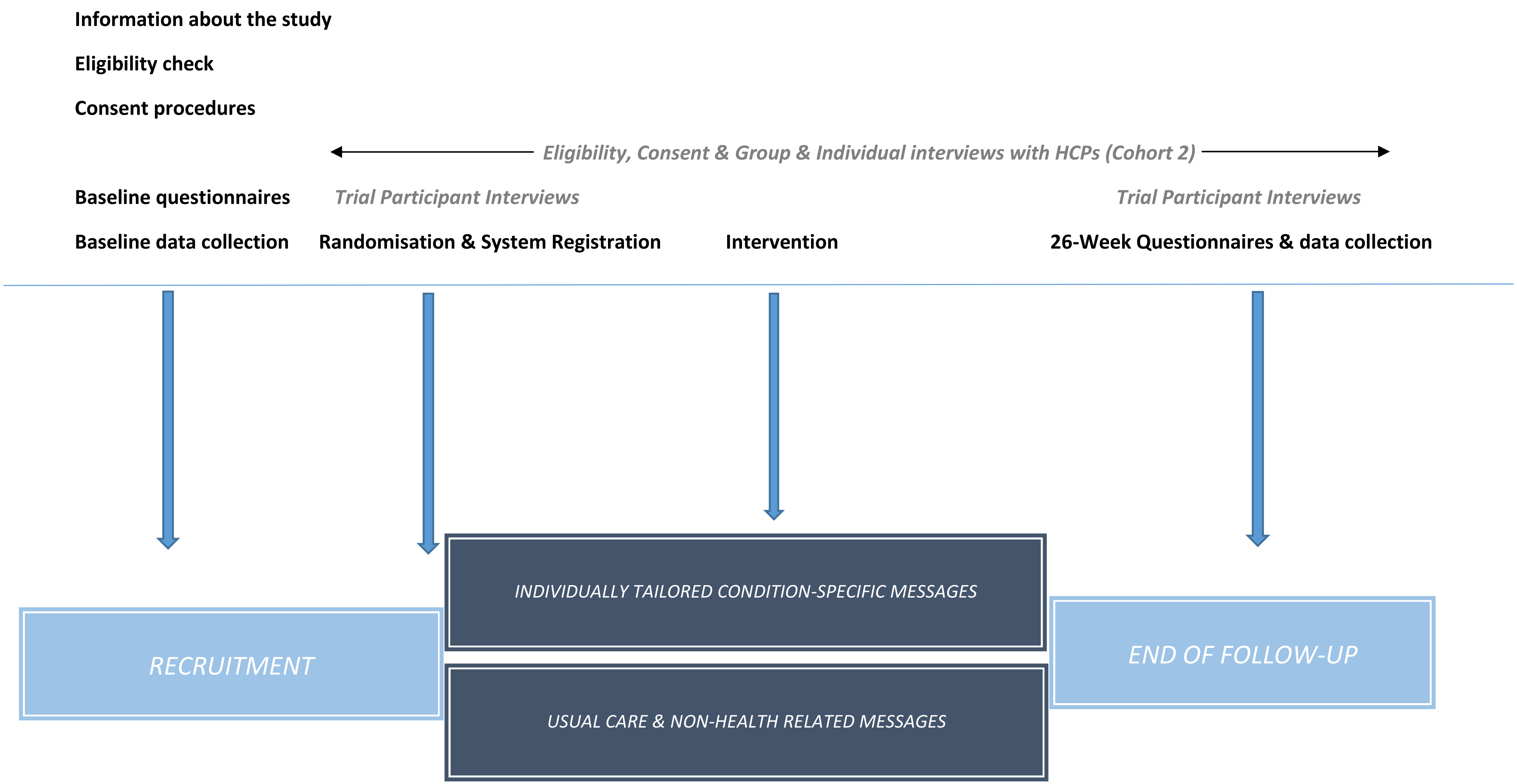

© Copyright: The University of Oxford and Oxford University Hospitals NHS Foundation Trust 2016

16 APPENDIX C: INTERVENTION COMPONENTS

16.1 Data Protection Particulars

The University of Oxford is the data controller by being the Sponsor of the SuMMiT-D Feasibility trial. The GP practices (also referred to as sites) recruiting for this trial will need to consent to EMIS sharing participant data with the Oxford University Hospitals NHS Foundation Trust, John Radcliffe Hospital servers in order to inform the text messages.
The data to be transferred includes: confirmation of data sharing consent, participant consent and clinical data previously covered in the protocol, specifically: Recorded clinic attendance, HbA1c measurements, height and weight, blood pressure measurements, total and HDL cholesterol and prescriptions issued. The data processing will cover the period from randomisation until the end of the 26-week (also referred to as six-month) follow-up period, during which participants will be receiving messages, and it will cover only participants that have been randomised to the intervention group.

16.2 Engineering: SuMMiT-D Feasibility backend SMS system

The backend SMS system includes a virtual server hosted within an NHS compliant network. The server will be password protected and accessible only by authorised research study team members. We have implemented a modular and scalable computer system that allows the management of the participants' registration to the SMS system. The system allows also the configuration of the participants' preferences and includes a withdrawal process. The system will be run a randomised SMS selection algorithm and communicates with the SMS engine (Esendex) to send messages to participants. The correct dispatch of messages is monitored and errors flagged to relevant research team members. Once successfully sent, messages are deleted from the SMS engine servers.

Communications between the computers logging into the backend system, and to and from the SMS engine, is encrypted using state-of-the-art TLS (Transport Layer Security) encrypted channels. The database containing participant information (such as the mobile phone number) will be password protected accessible only to the backend system and its authorised users.

The backend system database contains the digital medical summary needed to tailor the SMS. Access to this information will be allowed only to the automated system and for technical support and will not be available to logged users.

16.3 Health Psychology

The intervention has been designed to support people with type 2 diabetes encouraging and supporting them in developing a habit of taking their medication as intended and providing hints and tips to help them with other aspects of living with the condition. Participants register with an automated text message system. This system sends selected messages from a library of messages to participants. The type of messages sent are partly based on factors such as: the time since starting a new medication, smoking status, issue of prescription and automated text message participant feedback on whether participants want more or fewer of a particular type of message. Messages have been developed with a range of different underpinning behaviour techniques. Initially, messages will be sent randomly from among all the types of behaviour change technique, but participants' feedback, as noted above, will change the frequency and a type of messages.

Behaviour change (such as taking medication as prescribed) has been conceptualised as a process, from forming an intention to change the behaviour, acting on this intention, monitoring

© Copyright: The University of Oxford and Oxford University Hospitals NHS Foundation Trust 2016

and adjusting the action taken to maintain behaviour change, and finally to the behaviour becoming a habit without the need for effortful monitoring. This intervention targets the psychological constructs that are thought to influence each stage of this process. Measures will be taken during the feasibility trial to assess these proposed mechanisms of behaviour change (see Figure 1 for proposed theoretical model based on the Health Action Process Approach [10].

Message development
The tailored messages are either i) based on behaviour change techniques and beliefs and concerns related to taking medication or ii) based on other aspects of diabetes self-management.

The messages based on behaviour change techniques and beliefs and concerns have been developed through a 5-step process.

1) A rapid review was conducted to identify promising targets for behaviour change related to medication adherence. Some of these targets were psychological constructs e.g. attitude, or self-efficacy, others were particular beliefs and concerns people with type 2 diabetes had about taking their medication e.g. healthcare system related concerns or difficulties with side effects. Where possible, the identified targets were mapped onto recognised behaviour change techniques (BCTs), for example the BCT 'providing information about health consequences' is thought to influence attitude.

2) A workshop day was organised with experts in health psychology and healthcare professionals with experience of working with people with type 2 diabetes. On the day groups were asked to generate 160 character text messages either based on specific BCTs, or that addressed specific beliefs and concerns. Any BCTs that experts felt were inappropriate to deliver via text message were removed.

3) The messages were shown to people with type 2 diabetes in focus groups and both the overall idea of a text messaging system to support medication adherence and opinions about specific messages and approaches to behaviour change were gathered. The messages were re-worded according to participants' suggestions and suggestions made about the system overall were collated to influence further iterations of the system.

4) A sub-set of the messages were reviewed for acceptability by people with type 2 diabetes who completed a survey. Messages that were deemed difficult to understand were re-worded.

5) The messages have been formally reviewed through feedback from people with type 2 diabetes who have used the system, as well as by experts to ensure they represent the intended BCTs well.

The messages relating to aspects of diabetes management aside from medication adherence were drawn from credible online sources (Diabetes UK, NHS Choices, One You) following suggestions made by health care professionals and focus group participants. It has been agreed that the system would benefit from messages targeting aspects such as diet and physical activity. These messages have been reviewed by members of the research team.

Message scheduling
The messages are scheduled initially so that those BCTs that aim to increase motivation (and forming an intention) are sent alongside those that aim to support volitional processes (from the formation of an intention through to maintaining the behaviour). After 3 months, only the messages supporting volitional processes will be sent.

Throughout the intervention, participants can text keywords after any message to indicate whether they would like to see more messages like this, or fewer messages like this. In this way the content received will be tailored based on user preferences. Additional tailoring will be based

© Copyright: The University of Oxford and Oxford University Hospitals NHS Foundation Trust 2016

on facets such as sending messages related to smoking cessation only to those identified as current smokers and sending messages with multimedia links, only to those participants identified as using smartphones.

Participants will have the option to set the times they would like to receive the messages and being able to stop, pause and restart messages using automated interactive text messaging

© Copyright: The University of Oxford and Oxford University Hospitals NHS Foundation Trust 2016
CONFIDENTIAL

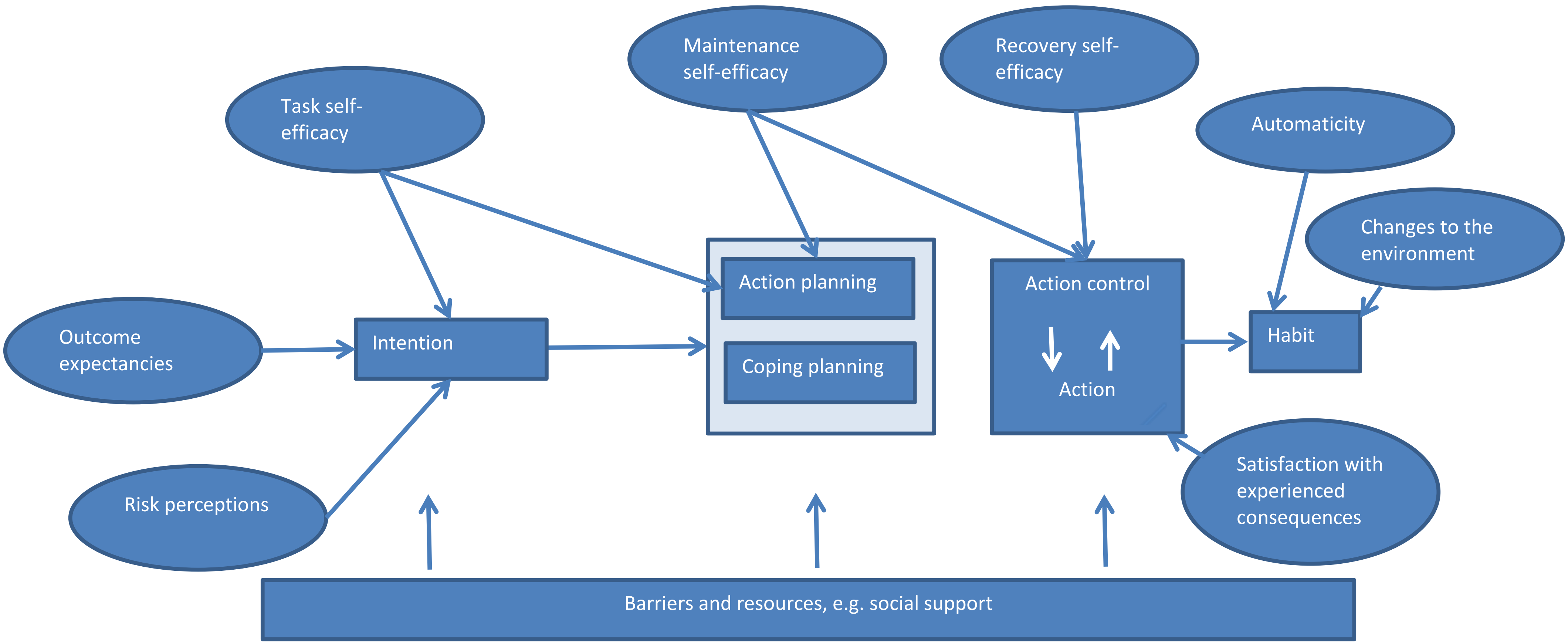

Figure 1: Proposed theoretical model based on the Health Action Process Approach

Schwarzer, R. Modeling health behavior change: How to predict and modify the adoption and maintenance of health behaviors. *Applied Psychology* 2008; **57,** 1–29

© Copyright: The University of Oxford and Oxford University Hospitals NHS Foundation Trust 2016
CONFIDENTIAL

17 APPENDIX D: AMENDMENT HISTORY

Amendment No.	Protocol Version No.	Date issued	Author(s) of changes	Details of Changes made

© Copyright: The University of Oxford and Oxford University Hospitals NHS Foundation Trust 2016
